# Factors affecting the electrocardiographic QT interval in malaria: A systematic review and meta-analysis of individual patient data

**DOI:** 10.1371/journal.pmed.1003040

**Published:** 2020-03-05

**Authors:** Xin Hui S. Chan, Yan Naung Win, Ilsa L. Haeusler, Jireh Y. Tan, Shanghavie Loganathan, Sompob Saralamba, Shu Kiat S. Chan, Elizabeth A. Ashley, Karen I. Barnes, Rita Baiden, Peter U. Bassi, Abdoulaye Djimde, Grant Dorsey, Stephan Duparc, Borimas Hanboonkunupakarn, Feiko O. ter Kuile, Marcus V. G. Lacerda, Amit Nasa, François H. Nosten, Cyprian O. Onyeji, Sasithon Pukrittayakamee, André M. Siqueira, Joel Tarning, Walter R. J. Taylor, Giovanni Valentini, Michèle van Vugt, David Wesche, Nicholas P. J. Day, Christopher L-H Huang, Josep Brugada, Ric N. Price, Nicholas J. White

**Affiliations:** 1 Mahidol-Oxford Tropical Medicine Research Unit, Faculty of Tropical Medicine, Mahidol University, Bangkok, Thailand; 2 Centre for Tropical Medicine and Global Health, Nuffield Department of Medicine, University of Oxford, Oxford, United Kingdom; 3 Health and Diseases Control Unit, Naypyidaw, Myanmar; 4 WorldWide Antimalarial Research Network, Centre for Tropical Medicine and Global Health, Nuffield Department of Medicine, University of Oxford, Oxford, United Kingdom; 5 University College London Great Ormond Street Institute of Child Health, London, United Kingdom; 6 Christ Church College, University of Oxford, Oxford, United Kingdom; 7 Singapore Armed Forces Medical Corps, Singapore; 8 Lao-Oxford-Mahosot Hospital-Wellcome Trust Research Unit, Vientiane, Lao PDR; 9 Division of Clinical Pharmacology, Department of Medicine, University of Cape Town, Cape Town, South Africa; 10 WorldWide Antimalarial Resistance Network, Cape Town, South Africa; 11 INDEPTH Network Secretariat, Accra, Ghana; 12 Department of Internal Medicine, Faculty of Clinical Sciences, College of Health Sciences, University of Abuja, Abuja, Nigeria; 13 Malaria Research and Training Center, Department of Epidemiology of Parasitic Diseases, Faculty of Pharmacy, University of Science Techniques and Technologies of Bamako, Bamako, Mali; 14 Department of Medicine, University of California San Francisco, San Francisco, California, United States of America; 15 Medicines for Malaria Venture, Geneva, Switzerland; 16 Department of Clinical Tropical Medicine, Faculty of Tropical Medicine, Mahidol University, Bangkok, Thailand; 17 Department of Clinical Sciences, Liverpool School of Tropical Medicine, Liverpool, United Kingdom; 18 Fundação de Medicina Tropical Dr Heitor Vieira Dourado, Manaus, Brazil; 19 Instituto Leônidas e Maria Deane (FIOCRUZ-Amazonas), Fundação Oswaldo Cruz, Manaus, Brazil; 20 Sun Pharmaceutical Industries Ltd, Gurgaon, Haryana, India; 21 Shoklo Malaria Research Unit, Mahidol-Oxford Tropical Medicine Research Unit, Faculty of Tropical Medicine, Mahidol University, Mae Sot, Thailand; 22 Faculty of Pharmacy, Obafemi Awolowo University, Ile-Ife, Nigeria; 23 The Royal Society of Thailand, Dusit, Bangkok, Thailand; 24 Instituto Nacional de Infectologia Evandro Chagas, Fundação Oswaldo Cruz, Rio de Janeiro, Brazil; 25 Corporate R&D Department, Alfasigma S.p.A., Rome, Italy; 26 Amsterdam University Medical Centers, Location Academic Medical Center, University of Amsterdam, Amsterdam, the Netherlands; 27 Certara, Princeton, New Jersey, United States of America; 28 Physiological Laboratory, University of Cambridge, Cambridge, United Kingdom; 29 Cardiovascular Institute, Hospital Clinic, University of Barcelona, Barcelona, Spain; 30 Global and Tropical Health Division, Menzies School of Health Research, Charles Darwin University, Darwin, NT, Australia; Burnet Institute, AUSTRALIA

## Abstract

**Background:**

Electrocardiographic QT interval prolongation is the most widely used risk marker for ventricular arrhythmia potential and thus an important component of drug cardiotoxicity assessments. Several antimalarial medicines are associated with QT interval prolongation. However, interpretation of electrocardiographic changes is confounded by the coincidence of peak antimalarial drug concentrations with recovery from malaria. We therefore reviewed all available data to characterise the effects of malaria disease and demographic factors on the QT interval in order to improve assessment of electrocardiographic changes in the treatment and prevention of malaria.

**Methods and findings:**

We conducted a systematic review and meta-analysis of individual patient data. We searched clinical bibliographic databases (last on August 21, 2017) for studies of the quinoline and structurally related antimalarials for malaria-related indications in human participants in which electrocardiograms were systematically recorded. Unpublished studies were identified by the World Health Organization (WHO) Evidence Review Group (ERG) on the Cardiotoxicity of Antimalarials. Risk of bias was assessed using the Pharmacoepidemiological Research on Outcomes of Therapeutics by a European Consortium (PROTECT) checklist for adverse drug events. Bayesian hierarchical multivariable regression with generalised additive models was used to investigate the effects of malaria and demographic factors on the pretreatment QT interval. The meta-analysis included 10,452 individuals (9,778 malaria patients, including 343 with severe disease, and 674 healthy participants) from 43 studies. 7,170 (68.6%) had fever (body temperature ≥ 37.5°C), and none developed ventricular arrhythmia after antimalarial treatment. Compared to healthy participants, patients with uncomplicated falciparum malaria had shorter QT intervals (−61.77 milliseconds; 95% credible interval [CI]: −80.71 to −42.83) and increased sensitivity of the QT interval to heart rate changes. These effects were greater in severe malaria (−110.89 milliseconds; 95% CI: −140.38 to −81.25). Body temperature was associated independently with clinically significant QT shortening of 2.80 milliseconds (95% CI: −3.17 to −2.42) per 1°C increase. Study limitations include that it was not possible to assess the effect of other factors that may affect the QT interval but are not consistently collected in malaria clinical trials.

**Conclusions:**

Adjustment for malaria and fever-recovery–related QT lengthening is necessary to avoid misattributing malaria-disease–related QT changes to antimalarial drug effects. This would improve risk assessments of antimalarial-related cardiotoxicity in clinical research and practice. Similar adjustments may be indicated for other febrile illnesses for which QT-interval–prolonging medications are important therapeutic options.

## Abstract 2

Xin Hui Chan and co-workers report an individual patient data meta-analysis on disease-specific heart rhythm alterations in malaria.

## Author summary

**Why was this study done?:**

- Prolongation of the electrocardiographic (ECG) QT interval is a widely used marker of the risk of developing abnormal heart rhythms, which can cause sudden death.
- Several antimalarial drugs are associated with QT interval prolongation, motivating systematic ECG safety monitoring in many malaria clinical studies.
- Malaria illness itself may also affect the heart and QT interval, but these disease effects are not well-understood.
- Interpretation of ECG changes after antimalarial treatment is complicated by the coincidence of peak antimalarial drug concentrations with malaria recovery.

**What did the researchers do and find?:**

- We performed a systematic review and meta-analysis of individual patient data to investigate the effect of malaria disease factors on the ECG QT interval.
- We included pretreatment data from 10,452 individuals (9,778 malaria patients and 674 healthy participants) from 43 studies in 20 countries.
- Malaria was associated with QT interval shortening and increased sensitivity of the QT interval to heart rate changes, and these effects increased with malaria severity.
- Body temperature increase (fever) was associated independently with clinically significant QT shortening.

**What do these findings mean?:**

- To improve cardiac safety assessments of antimalarial medicines, adjustment for malaria recovery- and defervescence-related QT interval prolongation is needed to avoid misattributing QT changes solely to drugs.
- These findings are relevant to drug developers, healthcare providers, clinical trialists, and policymakers who use QT interval safety data to decide which antimalarials to develop and use and at what dose.
- Further study of the QT interval in other infections and inflammatory disorders that cause fever and are treated with QT-prolonging medicines is recommended in order to understand the role of disease factors in QT interval changes.

## Introduction

Malaria remains the most important parasitic disease of humans. Over a thousand people—mostly children in Africa—still die of the disease every day. Decades of progress in prevention and control have now stagnated [1]. Strategic use of all available tools is essential to prevent reversal of these hard-won gains.

Antimalarial medicines are central to malaria control efforts. They are given both to prevent malaria and to treat it. The artemisinin-based combination therapies (ACTs) are now the gold standard oral treatment for malaria and the first-line antimalarial treatment in >80 malaria-endemic countries [2]. ACTs contain a rapidly acting artemisinin derivative combined with a more slowly eliminated partner drug. Most of the partner drugs in use and several of those in development are structurally related quinoline or quinoline-like compounds, some of which prolong the electrocardiographic QT interval. Drug-related QT interval prolongation is a widely used [3] yet nonspecific surrogate risk marker for repolarisation-related cardiotoxicity in the form of torsade de pointes, a potentially fatal polymorphic ventricular tachycardia. QT interval prolongation has been the most common reason for drug withdrawal and relabelling [4]. As part of ongoing safety assessments of population-based use of ACTs and other quinoline- or structurally related compound-containing combinations for malaria control and elimination in both acutely unwell patients and healthy people at risk of symptomatic disease, there has been renewed interest in the evaluation of antimalarial effects on the ECG to guide antimalarial selection and dosage [5–7].

Malaria is characterised by red blood cell parasitisation, fever, and anaemia [8]. Malaria illness itself may affect the heart and in particular the QT interval [9,10], although these disease effects and their possible interaction with other factors known to affect the QT interval [11] are not well-understood [12]. Because malaria illness and antimalarial drug concentrations change over the course of malaria treatment, it is important to characterise the independent contributions of disease and demographic factors on the QT interval in order to avoid misattributing changes solely to drug effects [12].

To address this, we conducted a systematic review and meta-analysis of individual patient data from malaria clinical trials to characterise the disease and demographic factors that independently affect the electrocardiographic QT interval in malaria.

## Methods

### Search strategy and selection criteria

We performed a systematic literature search on October 22, 2015 (updated on August 21, 2017) of the databases MEDLINE, Embase, and Global Health for primary clinical studies of the quinoline and structurally related antimalarials for malaria-related indications in which electrocardiograms (ECGs) were recorded before and after drug administration (Search Strategy in S1 Appendix). These published and additional unpublished studies were identified as part of the work of the World Health Organization (WHO) Evidence Review Group (ERG) on the Cardiotoxicity of Antimalarials [5].

Studies were eligible for inclusion in the review if they were prospective randomised-controlled trials or cohort studies published from 1988 onwards in which 5 or more participants were given a quinoline or structurally related antimalarial drug—amodiaquine, chloroquine, halofantrine, lumefantrine, mefloquine, piperaquine, primaquine, pyronaridine, or quinine—either as monotherapy or as part of an ACT. Studies that coadministered other drugs with QT-prolonging potential (e.g., azithromycin) as part of the trial intervention were excluded.

Study authors were contacted with a request for clinical study reports and protocols as well as anonymised individual patient-level data sets of the following prespecified variables identified from expert consultation [5]: age, weight, sex, body temperature, parasitaemia, haemoglobin or haematocrit, heart rate or RR interval duration, uncorrected QT interval duration, ECG abnormalities, and other cardiovascular adverse events. Studies were included in this meta-analysis if individual patient-level data were available for all requested variables from the screening or a baseline time point before antimalarial drug administration.

All included individual patient-level data were obtained in accordance with appropriate ethical approvals from countries and institutions of origin. Additional ethical approval for this systematic review and meta-analysis of fully anonymised individual patient data was not deemed necessary in keeping with University of Oxford Central University Research Ethics Committee guidance.

### Data extraction and standardisation

At least two independent reviewers (from XHSC, YNW, ILH, and SKSC) screened titles, abstracts, full texts, trial documentation, and anonymised data sets and agreed on study eligibility. From study publications, reports, and protocols, we extracted study-level characteristics, including location, antimalarial treatment indication, inclusion and exclusion criteria, temperature measurement method, and ECG measurement methodology (Data Extraction in S1 Appendix), into a standardised database. Where required, trial registry records and study investigators were consulted for further information.

Manual data entry was undertaken for data sets available only in printed format. Once digitised, individual patient-level data sets were converted into a standard file format using Stat/Transfer [13] version 13.3, then standardised and checked according to a prespecified data dictionary (Data Standardisation in S1 Appendix). For studies of repeated treatments, only data from the first treatment episode were extracted. Individual patient records were excluded if data for any requested variables were missing at the selected time point before drug administration (Data Integrity Checks in S1 Appendix).

### Data analysis

We performed Bayesian hierarchical multivariable regression with generalised additive models. The QT interval was the response variable, and individual study ID was the varying intercept. The square-root–transformed RR interval ($\sqrt{RR}$), sex, body temperature, and malaria type/antimalarial treatment indication were the linear predictors. Age was modelled with separate smooths for females and males because of the known sex-hormone–related QT interval changes around puberty [11]. Weight was omitted because of its collinearity with age in a predominantly paediatric population. Haemoglobin was considered an intermediate variable and omitted. Variable selection was based on directed acyclic graphs of proposed causal relationships among collected variables (Fig B in S1 Appendix) identified from literature review and expert consultation [5].

Four models were fitted to the data, in which different combinations of malaria-disease–related terms were added to known factors affecting the QT interval: the first contained only heart rate and demographic terms, the second added to the first a term for body temperature, the third included a further term for malaria type (species, severity), and the fourth added to the third an interaction term for malaria type and $\sqrt{RR}$. We carried out Pareto smoothed importance-sampling leave-one-out cross-validation for model comparison (Data Analysis in S1 Appendix). We did not publish or preregister this analysis plan.

As sensitivity analyses, we compared the model with the best expected predictive performance to a model with an additional binary variable for whether a participant was in a study that excluded at screening individuals with one or more torsade de pointes risk factors, a model with a linear predictor for haemoglobin, and another model with a cube root (Fridericia-like) instead of square root (Bazett-like) transformation of the RR interval. In the subgroup of malaria patients only, we added a linear predictor for log parasitaemia to the best model (Data Analysis in S1 Appendix).

All statistical analyses and data visualisation were done in R [14] version 3.5.0. Bayesian regression was done using the brms [15] package version 2.6.0 and the probabilistic programming language Stan [16] version 2.18.0.

The risk of bias of individual studies at the outcome level was assessed using the PROTECT [17] checklist for systematic reviews on adverse drug events.

## Results

**Fig 1:**
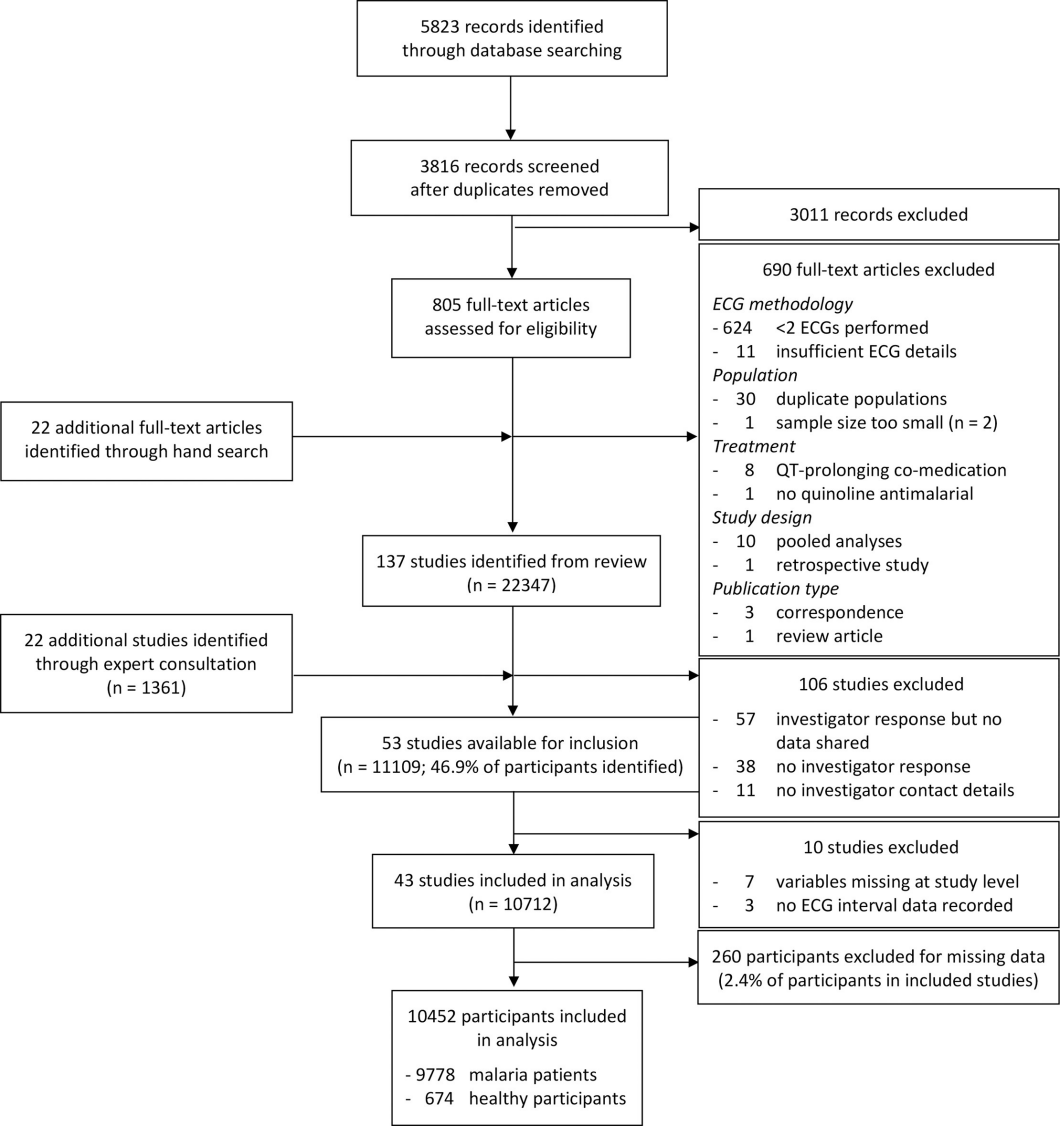
Study selection flow chart. ECG, electrocardiogram.

Individual patient-level data were sought from 159 clinical studies (137 published and 22 unpublished at the time of the literature search). Data from 11,109 participants in 53 studies were shared, of which data from 10,452 participants in 43 studies (28 published [18–45], 5 subsequently published [46–50], and 10 unpublished) were suitable for inclusion in the meta-analysis (Fig 1 and Tables A and B in S1 Appendix).

Overall, 93.6% (9,778/10,452) of included individuals had microscopy-confirmed *Plasmodium falciparum* or *P*. *vivax* malaria, of whom 89.7% (8,769/9,778) had uncomplicated *P*. *falciparum* mono- or mixed infection (Table 1). The remaining 674 individuals were healthy participants, the majority of whom (78.8%; 531/674) were enrolled in healthy volunteer pharmacokinetic studies (Table 1). The median age of the 10,452 included individuals was 13.3 years (interquartile range [IQR] 4.6–26.0; range 6 months to 84 years), with 26.8% (2,803) aged 5 to <15 years and 26.3% (2,751) aged <5 years. The healthy group were almost entirely adult participants from Europe, North America, and urban Asia and Africa, while malaria patients were more likely to be children or adolescents from rural Africa, Asia, and South America and to have fever (temperature ≥ 37.5°C), anaemia, and tachycardia (Table 1 and Figs C–E in S1 Appendix).

**Table 1:** Demographics and population characteristics.

	Healthy Participants (*n* = 674)	Malaria Patients (*n* = 9,778)	Overall (*n* = 10,452)
**Antimalarial Treatment Indication**			
Severe/complicated malaria		343 (3.5%)	343 (3.3%)
Uncomplicated malaria		9,435 (96.5%)	9,435 (90.3%)
*P*. *falciparum* mono- or mixed infection		8,769 (89.7%)	8,769 (83.9%)
*P*. *vivax* monoinfection		666 (6.8%)	666 (6.4%)
IPT	143 (21.2%)		143 (1.4%)
Pregnancy (IPTp)	125 (18.5%)		125 (1.2%)
Infancy (IPTi)	18 (2.7%)		18 (0.2%)
Healthy volunteer pharmacokinetics	531 (78.8%)		531 (5.1%)
**Age (years)**			
Median (IQR)	28.9 (23.0–37.0)	12.1 (4.2–24.5)	13.3 (4.6–26.0)
<15	18 (2.7%)	5,536 (56.7%)	5,554 (53.1%)
<1	18 (2.7%)	193 (2.0%)	211 (2.0%)
1 to <5	0	2,540 (26.0%)	2,540 (24.3%)
5 to <15	0	2,803 (28.7%)	2,803 (26.8%)
≥15	656 (97.3%)	4,242 (43.4%)	4,898 (46.9%)
≥35	209 (31.0%)	1,296 (13.3%)	1,505 (14.4%)
≥50	0	40 (0.41%)	40 (0.38%)
**Sex**			
Female	343 (50.9%)	3,909 (40.0%)	4,252 (40.7%)
Pregnant	125 (18.5%)	9 (0.09%)	134 (1.3%)
Male	331 (49.1%)	5,869 (60.0%)	6,200 (59.3%)
**Temperature (°C)**			
Mean (SD)	36.8 (0.4)	38.2 (1.1)	38.2 (1.1)
≥37.5	23 (3.4%)	7,147 (73.1%)	7,170 (68.6%)
**Parasitaemia (parasites/μL)**			
Median (IQR)	N/A	14,080 (2,851–45,219)	14,080 (2,851–45,219)
≥10,000	N/A	5,500 (56.2%)	5,500 (52.6%)
≥50,000	N/A	2,191 (22.4%)	2,191 (20.9%)
≥100,000	N/A	905 (9.3%)	905 (8.7%)
≥250,000	N/A	175 (1.8%)	175 (1.7%)
**Heart Rate (beats per minute)**			
Mean (SD)	68 (17)	108 (30)	106 (31)
≥140	0	1,537 (15.7%)	1,537 (14.7%)
120–139	16 (2.4%)	1,603 (16.4%)	1,619 (15.5%)
100–119	22 (3.3%)	2,356 (24.1%)	2,378 (22.8%)
80–99	79 (11.7%))	2,560 (26.2%)	2,639 (25.2%)
60–79	320 (47.4%)	1,537 (15.7%)	1,857 (17.8%)
<60	237 (35.2%)	185 (1.9%)	422 (4.0%)
**Torsade de Pointes Risk Factors**			
Excluded from the individual study	669 (99.3%)	6,964 (71.2%)	7,633 (73.0%)
Not excluded from the individual study	5 (0.7%)	2,814 (28.8%)	2,819 (27.0%)
**Geographical Region**			
Africa	172 (25.5%)	6,363 (65.0%)	6,535 (62.5%)
Asia	147 (21.8%)	3,065 (31.3%)	3,212 (30.7%)
Americas	15 (2.2%)	350 (3.6%)	365 (3.5%)
Europe	340 (50.4%)	0	340 (3.3%)

**Abbreviations:** ECG, electrocardiogram; IPT, intermittent preventive therapy; IQR, interquartile range; N/A, not applicable; SD, standard deviation.

60.5% (26/43) of studies listed as exclusion criteria one or more risk factors for torsade de pointes, such as a personal or family history of clinically significant arrhythmias, pre-existing conditions or concomitant medications that prolong the QT interval or increase antimalarial drug concentrations, a baseline corrected QT interval of more than 450 milliseconds, and electrolyte imbalances including hypokalaemia and hypomagnesaemia. These 26 studies that excluded patients with risk factors for torsade de pointes enrolled 73% (7,633/10,452) of individuals providing data.

Almost all (99.3%; 10,381/10,452) participants, including all malaria patients, had ECG intervals measured manually by cardiologists (75.3%; 7,872/10,452) or other trained personnel. In addition, 68.6% (7,170/10,452) of participants in 30.2% (13/43) of studies had ECGs sent to a centralised facility where specialist staff read ECGs, and in the remainder, ECGs were read at the study site. Only two studies, both of piperaquine in healthy volunteers [48,51], had 24-hour continuous ECG recordings at baseline (Tables C and D in S1 Appendix).

None of the 10,452 participants included had a baseline uncorrected QT interval of more than 500 milliseconds.

Compared to included studies, a higher proportion of excluded studies were conducted before 2007, did not specifically exclude torsade de pointes risk factors, and had unclear or high risk of bias (Tables E and F in S1 Appendix), reflecting quality of measurement and reporting methods of safety outcomes. The characteristics of included and excluded studies were otherwise comparable (Table E in S1 Appendix). As with the excluded studies, most of the 260 participants who were excluded for missing data were malaria patients in studies conducted before 2007 with available characteristics similar to those of the included population (Table G in S1 Appendix). There were no cases of sudden cardiac death, life-threatening ventricular tachyarrhythmias (ventricular fibrillation or ventricular tachycardia), or torsade de pointes documented for any of the 23,708 participants in the 159 studies from which individual patient-level data were sought.

### Effect of malaria on the QT interval

Adjustment for malaria disease variables of body temperature and malaria type improved model performance (Table H in S1 Appendix). Results are presented from the best model, which had heart rate (as $\sqrt{RR}$), age, sex, body temperature, and malaria type (both as an independent term and an interaction term with heart rate) as predictors.

**Fig 2:**
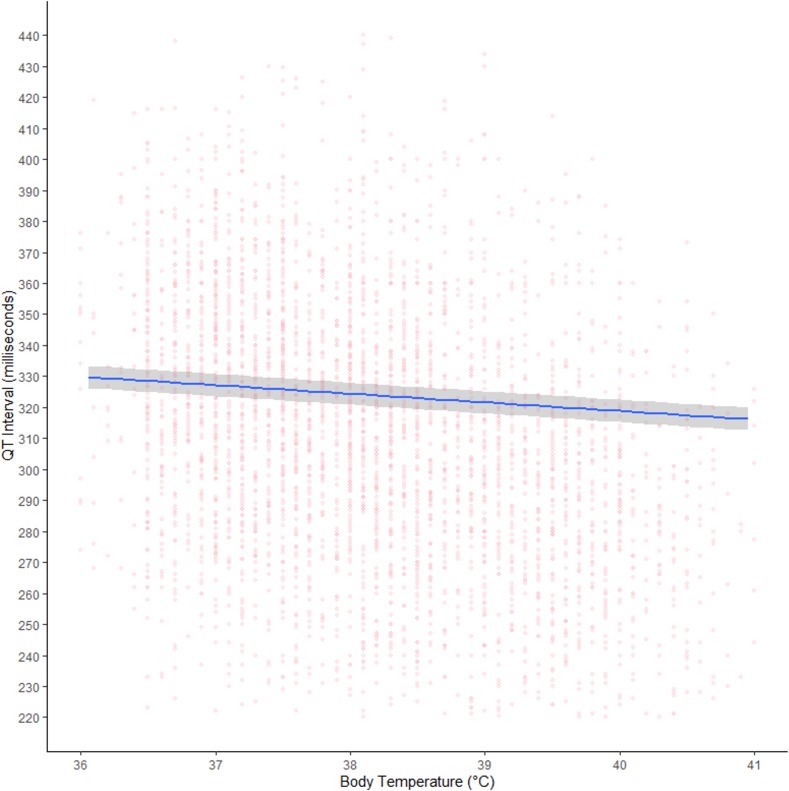
Body temperature and the QT interval in malaria. Independent effect of body temperature on the QT interval from hierarchical generalised additive model adjusting for heart rate/RR interval (as $\sqrt{RR}$), age, sex, malaria type, and individual study. Shaded area represents 95% CIs, and circles represent original data points without adjustment. CI, credible interval.

**Fig 3:**
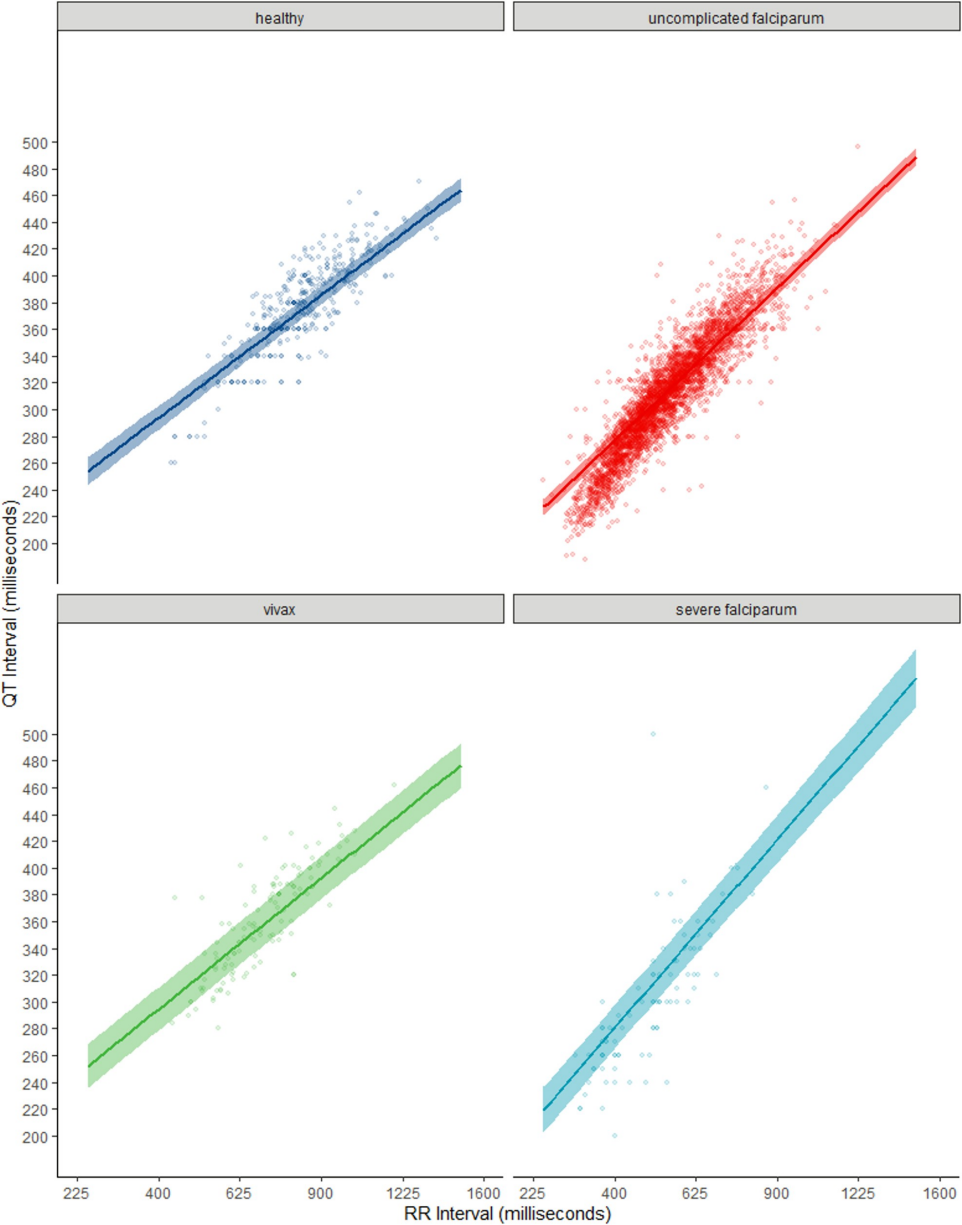
Malaria type, RR interval, and the QT interval. Interaction between malaria type and the RR interval (on the square root scale) and conditional effect on the QT interval (on the linear scale) from a hierarchical generalised additive model adjusting for age, sex, body temperature, and individual study. Shaded areas represent 95% CIs, and circles represent original data points without adjustment. CI, credible interval.

From the meta-analysis of all included participants (*n* = 10,452), body temperature had an independent effect on the QT interval, with a mean shortening of the QT interval by 2.80 milliseconds (95% credible interval [CI]: 2.42 to 3.17) per 1°C rise in temperature (Table 2 and Fig 2). When compared to healthy participants (*n* = 674) and adjusting for other predictors, QT shortening increased with malaria severity: patients with severe malaria (*n* = 343) had the shortest QT intervals (mean difference: −110.89 milliseconds; 95% CI: −140.38 to −81.25), followed by patients with uncomplicated falciparum malaria (*n* = 8,769) (mean difference: −61.77 milliseconds; 95% CI: −80.71 to −42.83). Patients with uncomplicated vivax malaria (*n* = 666) also had shorter QT intervals than healthy participants, but the 95% CI included zero (mean difference: −11.77 milliseconds; 95% CI −37.30 to 14.72). Sensitivity of the QT interval to changes in heart rate also increased with malaria severity: the additional increase in the QT interval per unit increase of $\sqrt{RR}$ (i.e., with decreasing heart rate) was higher in severe malaria patients (mean difference: 4.89 milliseconds; 95% CI: 3.85 to 5.91) than patients with uncomplicated falciparum malaria (mean difference: 2.24 milliseconds; 95% CI: 1.65 to 2.83). These values compared with a mean increase of 9.16 milliseconds (95% CI: 8.59 to 9.73) in healthy participants. Again, uncomplicated vivax malaria patients had a slightly larger increase in the QT interval with decreasing heart rate than healthy participants, but the 95% CI contained zero (mean difference: 0.62 milliseconds; 95% CI: −0.11 to 1.34) (Table 2 and Fig 3).

**Table 2:** Factors affecting the QT interval in malaria.

Predictor	Number of Participants	Estimate (95% CI)/Smooth Description	Clinically Significant?	Improved Model?^†^
$\sqrt{RR}$ interval, per √millisecond increase* (healthy participants)	10,452	9.16 (8.59, 9.73) milliseconds	Yes	N/A
$\sqrt{RR}$ interval, per √millisecond increase* (by malaria type versus healthy participants)	10,452		Yes	Yes
Healthy participants	674	Reference		
Uncomplicated vivax malaria	666	0.62 (−0.11, 1.34) milliseconds		
Uncomplicated falciparum malaria	8,769	2.24 (1.65, 2.83) milliseconds		
Severe/complicated malaria	343	4.89 (3.85, 5.91) milliseconds		
Age	10,452		Yes	N/A
Female	4,252	Lengthens by approximately 8 milliseconds over childhood, then lengthens more gradually by another approximately 5 milliseconds in adulthood		
Male	6,200	Lengthens by approximately 8 milliseconds over childhood, then shortens by approximately 10 milliseconds around puberty before gradually lengthening by approximately 10 milliseconds in adulthood		
Sex	10,452		Yes	N/A
Female	4,252	Reference		
Male	6,200	−4.22 (-5.00, −3.43) milliseconds		
Body temperature, per 1°C increase	10,452	−2.80 (-3.17, −2.42) milliseconds	Yes	Yes
Malaria Type	10,452		Yes	Yes
Healthy participants	674	Reference		
Uncomplicated vivax malaria	666	−11.77 (−37.30, 14.72) milliseconds		
Uncomplicated falciparum malaria	8,769	−61.77 (−80.71, −42.83) milliseconds		
Severe/complicated malaria	343	−110.89 (−140.38, −81.25) milliseconds		

*Electrocardiographic RR interval in milliseconds = 60,000/(heart rate in beats per minute).

^†^Improved expected predictive accuracy as estimated by the standard error of the difference in expected log predictive density. Multivariable regression results from hierarchical generalised additive model. **Abbreviations:** CI, credible interval; N/A, not applicable.

**Fig 4:**
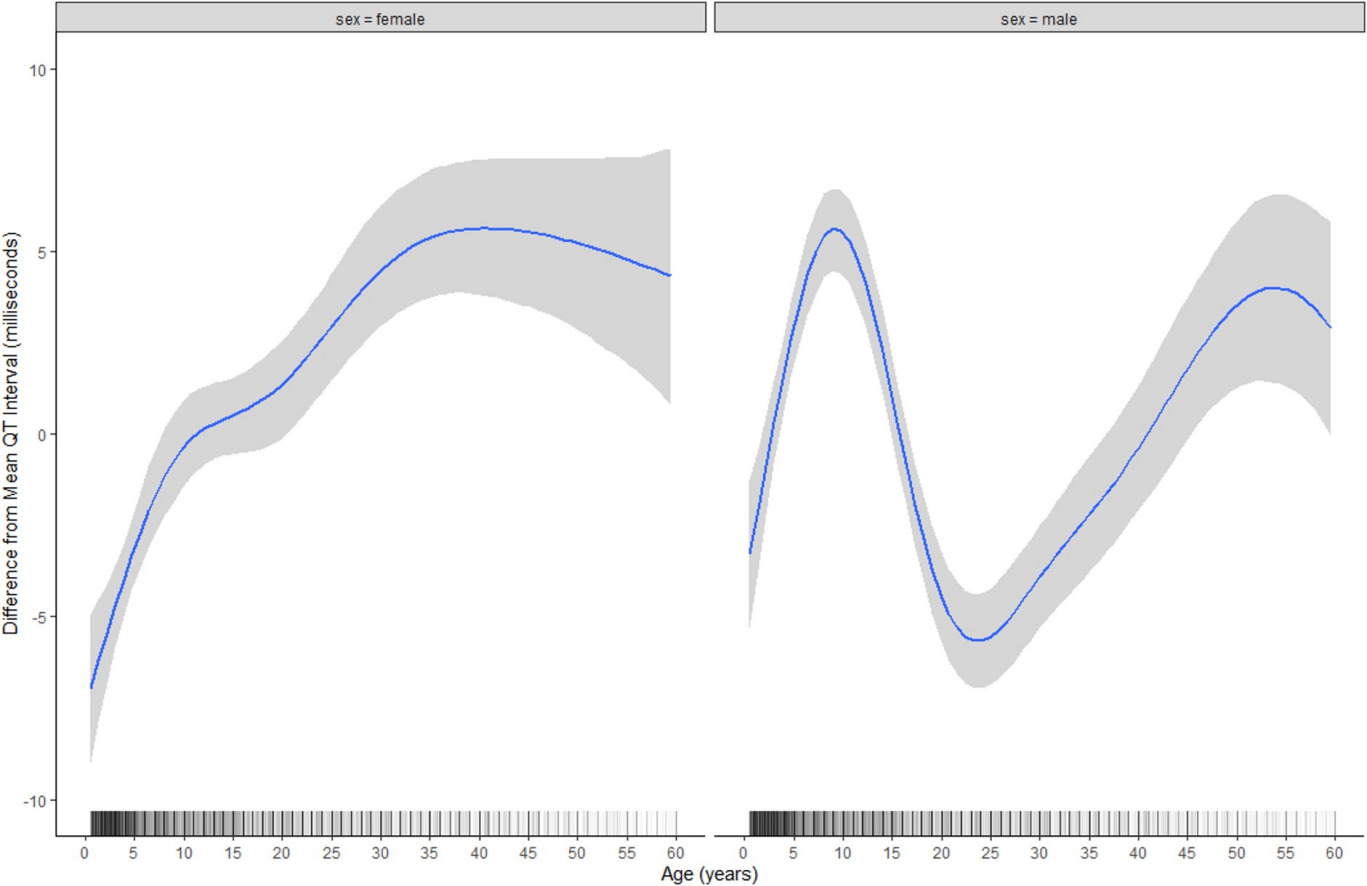
Age, sex, and the QT interval in malaria. Interaction between age and sex, and conditional effect on the QT interval, from a hierarchical generalised additive model adjusting for heart rate/RR interval (as $\sqrt{RR}$), malaria type, body temperature, and individual study. Shaded areas represent 95% CIs, and rug marks represent age distribution of original data points. CI, credible interval.

The demographic variables of age and sex had clinically significant effects: the QT interval lengthened by a mean of approximately 8 milliseconds over childhood before shortening by approximately 10 milliseconds in males, but not in females, around puberty, then gradually lengthened by approximately 5–10 milliseconds in both sexes over adulthood, although there were few data for participants aged ≥50 years (*n* = 40); males (*n* = 6,200) also had overall shorter QT intervals than females (*n* = 4,252) (mean difference: −4.21 milliseconds; 95% CI: −4.99 to −3.44) (Table 2 and Fig 4).

From this model, a 25-year–old male patient with uncomplicated falciparum malaria admitted with a heart rate of 100 beats per minute and a temperature of 38.5°C, whose heart rate slows to 60 beats per minute and who defervesces to a temperature of 36.5°C in recovery, would be predicted to have a 22 millisecond or 25% greater QT interval lengthening than an age- and sex-matched healthy afebrile participant with the same heart rate reduction independent of any drug treatment (Table 3).

**Table 3:** Predicted QT intervals at baseline and in recovery from malaria and fever.

	Healthy	Uncomplicated Vivax	Uncomplicated Falciparum	Severe Malaria
QT interval at baseline, milliseconds (95% PI) [HR = 100 bpm]	329 (285–371) [T = 36.5°C]	327 (281–370) [T = 38.5°C]	317 (273–358) [T = 38.5°C]	332 (287–377) [T = 38.5°C]
QT interval in recovery, milliseconds (95% PI) [HR = 60 bpm]	394 (351–435) [T = 36.5°C]	403 (356–446) [T = 36.5°C]	404 (360–445) [T = 36.5°C]	438 (392–484) [T = 36.5°C]
QT lengthening from baseline, milliseconds	65	76	87	106
Additional QT lengthening from baseline compared to healthy participant, milliseconds	0	11	22	41
Malaria-related QT lengthening from baseline, %	0	14	25	39

**Abbreviations:** bpm, beats per minute; HR, heart rate; PI, prediction interval; T, body temperature. Predicted values for a 25-year–old male from a hierarchical generalised additive model adjusting for heart rate/RR interval (as $\sqrt{RR}$), age, sex, malaria type, body temperature, and individual study effects.

### Sensitivity analyses

The QT intervals of participants in studies that screened for and excluded individuals with torsade de pointes risk factors (*n* = 7,633) were not significantly different from those in studies without documented risk factor screening (*n* = 2,819) (mean difference: −0.78 milliseconds; 95% CI −9.69 to 7.88) once other predictors had been adjusted for. There were no clinically significant changes in predictor estimates when a cube root (Fridericia-like) instead of square root (Bazett-like) transformation of the RR interval was used (Tables I–L in S1 Appendix).

The small effects associated with haemoglobin (−0.51 milliseconds per g/dL increase; 95% CI: −0.72 to −0.30) and parasitaemia (0.65 milliseconds per 10-fold increase in parasite density; 95% CI: 0.17 to 1.14) were of unclear clinical significance, and these additional terms did not improve model performance (Tables M–P, S1 Appendix).

## Discussion

To our knowledge, this is the most extensive study to date of the factors affecting the electrocardiographic QT interval in malaria. We pooled individual patient data before treatment from 10,452 adults and children (9,778 malaria patients, including 343 with severe disease, and 674 healthy participants) in antimalarial drug trials identified through a comprehensive systematic review and expert consultation. This allowed formal evaluation of the independent effects of malaria disease (type, temperature, and parasitaemia) and patient demographics (age and sex) on the QT interval without confounding from antimalarial drug effects through meta-analysis using hierarchical generalised additive models. Of these, malaria type, body temperature, age, and sex were found or confirmed to have clinically relevant effects on the QT interval.

Malaria has important effects on the QT interval that are proportional to disease severity: the marked QT interval shortening and increased sensitivity to changes in heart rate seen in malaria are greater in severe than uncomplicated disease and greater in *P*. *falciparum* than in *P*. *vivax* infection. These effects occur independently of temperature and parasitaemia, suggesting further unmeasured disease factors may be responsible. Possible candidates include increasing stimulation of the sympathetic nervous system and acid-base abnormalities resulting from microvascular sequestration with increasing malaria severity. Parasite density is a poor predictor of sequestered parasite burden in falciparum malaria [8]. Higher levels of proinflammatory cytokines such as interleukin-6 during acute infection in less immune populations [52] and in severe malaria [53] may also contribute through inhibition of cardiomyocyte ion channel function [54,55]. Yet, for a disease with a wide range of systemic complications causing multiple organ dysfunction, the heart is relatively spared in acute malaria: clinically significant arrhythmias are rare [7], and even in severe malaria, in which there may be extensive sequestration of parasitised erythrocytes in the myocardial microvasculature, cardiac performance is maintained [56,57].

Fever, a cardinal sign of malaria illness, was found to have an independent effect on the QT interval: the QT interval shortens as body temperature increases and lengthens correspondingly as temperature decreases. In other words, fever shortens the QT interval, and recovery from fever lengthens it. This is supported by the only prospective study, to our knowledge, of fever and the QT interval [58] without confounding from drug treatment, which measured ECGs in 27 otherwise healthy young Finnish male soldiers before and after self-limiting uncomplicated febrile illness of bacterial, viral, or undefined aetiology. That study found that the QT interval was significantly shorter during fever than after recovery (measurement of QT intervals at specific heart rates of 60, 80, and 100 beats per minute obviated the need for heart rate correction). Further prospective evaluation of the QT interval in febrile illness would be useful to assess whether the pyrexial shortening of the QT interval extends to fevers of other aetiologies [59], particularly because in vitro evidence indicates that the risk of drug-induced long QT syndrome is temperature-dependent [60]. This would be especially relevant for infectious diseases that present with fever and for which QT-interval–prolonging medications (e.g., macrolide and quinolone antibiotics) are also important therapeutic options.

Fever is known to unmask and trigger potentially life-threatening arrhythmias in individuals with inherited cardiac channelopathies. The arrhythmogenicity of fever is well established for Brugada syndrome [61], a leading cause of sudden unexplained death in young Southeast Asian men. It has also been observed in cases of type 2 congenital long QT syndrome with temperature-dependent phenotypes of human ether-à-go-go–related gene potassium channel mutations in which fever paradoxically prolonged instead of shortened the QT interval [62]. In our meta-analysis, there were no potentially life-threatening arrhythmias observed despite 73.1% (7,147/9,778) of participants with malaria being febrile. This may in part be because 71.2% (6,964/9,778) of malaria patients were enrolled in studies that excluded at screening individuals with torsade de pointes risk factors such as congenital channelopathies and concomitant medications known to prolong the QT interval. It may also be that the QT interval shortening seen during malarial fever could have a protective effect against ventricular arrhythmias.

Our meta-analysis further confirms established relationships between the QT interval and the demographic factors of age and sex are relevant in malaria. The QT interval does not exhibit a sex difference in childhood [10] until around puberty, when it shortens in males but not females [28], then lengthens gradually in adulthood in males more than females [11]. This difference is thought to result from pubertal changes in sex hormone levels, although the underlying mechanisms are not fully understood [11]. Postpubertal females have a higher risk of torsade de pointes [63] but a lower risk of sudden cardiac death at all ages [64]. Adjustment for sex-related differences when evaluating the QT interval in postpubertal individuals should be considered.

The flaws of commonly used QT correction factors adjusting for heart rate through proportional scaling with power functions (e.g., Bazett’s [65] and Fridericia’s [66] formulae) are well known and have been evaluated in large studies of healthy adults [67,68]. Reasons for their inadequacy in the healthy adult population include distorted correction, with substantial residual heart rate dependence of the corrected QT interval (particularly at extreme heart rates), and failure to account for sex differences in QT interval dynamics [67]. Moreover, these correction factors do not address additional confounding from disease effects on the QT interval that are independent of heart rate. In our meta-analysis, we performed regression analyses [67] with heart rate (as the square-root–transformed RR interval), age, sex, and malaria disease variables (type, temperature, and parasitaemia) as predictors. This approach avoided the problems of proportional scaling for heart rate correction by retaining an intercept term and investigated any independent additive effects of malaria disease on the QT interval with multivariable regression. Because malaria is associated with high heart rates, the distortions produced from proportional scaling for heart rate correction seen in healthy participants would be even more pronounced in malaria patients. The effects of malaria severity and temperature seen in our analyses further suggest that meaningful comparison of corrected QT intervals between healthy participants and malaria patients or even between repeated measurements from the same individual comparing acute malaria with recovery may be difficult without appropriate adjustment for these malaria disease effects.

Our study has several potential limitations. First, data were available from only about half of the participants identified. However, the included studies were more likely to have a low risk of bias than excluded studies because most were conducted in the last decade and had more comprehensive measurement and reporting methods, reflecting the increased regulatory interest in the cardiac safety of antimalarials. Second, it was not possible to assess directly the effects of other factors known to contribute to the intrinsic variability of the QT interval such as circadian rhythm, activity level, postural changes, and food ingestion [3], as well as those that may alter cardiomyocyte electrophysiology during systemic illness such as inflammatory biomarkers [54], because these data are not usually collected in malaria clinical trials. Most hospitalised malaria patients are supine and anorexic. Third, we have considered interindividual measurements from a single time point before drug administration, an approach that allowed us to consider data from a large number of patients with malaria and a smaller number of healthy participants without confounding from drug therapy. While assessments of repeated measurements from patients undergoing treatment for malaria with drugs not known to prolong the QT interval would be valuable, these data are few [7] and were not available to us.

Evaluation of QT interval prolongation after treatment with quinoline and structurally related antimalarials has been the major motivation for ECG monitoring in malaria. ECG monitoring is an operational challenge in the resource-limited settings where malaria is endemic and would severely limit the use of any drug for which monitoring is mandatory. In this large study of the QT interval in malaria, we have found malaria shortens the QT interval and increases its sensitivity to changes in heart rate. These differences are greater in severe malaria [9]. In addition, fever shortens the QT, and recovery from fever lengthens it. In acute uncomplicated malaria, there is usually an irregular fever with an appropriate rise in heart rate. As the illness and fever resolve, the heart rate declines to normal. This often coincides with the highest blood concentrations of slowly eliminated quinoline antimalarial drugs, which usually peak on the third day of treatment. The QT interval lengthening seen with recovery from malaria after antimalarial therapy results from resolution of disease effects in addition to any drug effects. Comparisons of predrug QT interval measurements with those at peak drug concentrations in malaria studies should take into account malaria- and fever-recovery–related QT lengthening; this would avoid excessive attribution of QT prolongation to the antimalarial treatment and improve risk assessments of potential antimalarial-related cardiotoxicity. This could avoid unnecessary discontinuation of new drug development and reduce the need for unnecessary adjustment or withdrawal of antimalarial treatment in response to malaria-related QT changes during research trials and clinical care. Similar adjustments may also be indicated for other febrile illnesses for which QT-interval–prolonging medications are important therapeutic options.

## Supporting information

S1 Checklist PRISMA-IPD checklist. PRISMA-IPD, Preferred Reporting Items for Systematic Reviews and Meta-analyses of Individual Patient Data.
(DOCX)

S1 Appendix Supplementary methods and results. (DOCX)

## Abbreviations

ACT: artemisinin-based combination therapy
CI: credible interval
ECG: electrocardiogram
EQUATOR: Enhancing the Quality and Transparency of Health Research
ERG: Evidence Review Group
IQR: interquartile range
PROTECT: Pharmacoepidemiological Research on Outcomes of Therapeutics by a European Consortium
WHO: World Health Organization
