# Supplementary material for: Factors affecting the electrocardiographic QT interval in malaria: A systematic review and meta-analysis of individual patient data
Source: PLoS Med. 2020 Mar 5;17(3):e1003040. doi: 10.1371/journal.pmed.1003040 (PMC7058280; doi:10.1371/journal.pmed.1003040)
Supplement: S1 Appendix — (DOCX) [file pmed.1003040.s002.docx]

**Appendix**

**Factors Affecting the Electrocardiographic QT Interval in Malaria:
A Systematic Review and Meta-analysis of Individual Patient Data**

Xin Hui S Chan, Yan Naung Win, Ilsa L Haeusler, Jireh Y Tan, Shanghavie Loganathan,
Sompob Saralamba, Shu Kiat S Chan, Elizabeth A Ashley, Karen I Barnes, Rita Baiden, Peter U Bassi,
Abdoulaye Djimdé, Grant Dorsey, Stephan Duparc, Borimas Hanboonkunupakarn,
Feiko O ter Kuile, Marcus VG Lacerda, Amit Nasa, François H Nosten, Cyprian O Onyeji,
Sasithon Pukrittayakamee, André M Siqueira, Joel Tarning, Walter RJ Taylor,
Giovanni Valentini, Michèle van Vugt, David Wesche, Nicholas PJ Day,

Christopher L-H Huang, Josep Brugada, Ric N Price, Nicholas J White

Table of Contents

[Supplementary Methods 5](#_Toc30471382)

[Search Strategy 5](#_Toc30471383)

[Study-Level Data Extraction 6](#_Toc30471384)

[Individual Patient-Level Data Standardisation 7](#_Toc30471385)

[ECG Intervals 7](#_Toc30471386)

[RR Interval 7](#_Toc30471387)

[QT/QTc interval 7](#_Toc30471388)

[Demographics 7](#_Toc30471389)

[Age 7](#_Toc30471390)

[Weight 7](#_Toc30471391)

[Vital Signs 7](#_Toc30471392)

[Temperature 7](#_Toc30471393)

[Laboratory Parameters 8](#_Toc30471394)

[Parasitaemia 8](#_Toc30471395)

[Haemoglobin 8](#_Toc30471396)

[Individual Patient-Level Data Integrity Checks 8](#_Toc30471397)

[Data Analysis 9](#_Toc30471398)

[Exploratory Analyses 9](#_Toc30471399)

[Variable Selection 9](#_Toc30471400)

[Model Formulation 9](#_Toc30471401)

[Model Priors 9](#_Toc30471402)

[Model Diagnostics & Posterior Predictive Checks 9](#_Toc30471403)

[Model Checking and Comparison 10](#_Toc30471404)

[Figure A: Principal Component Analysis Biplots of Factors Affecting the QT Interval in Malaria 11](#_Toc30471405)

[Figure B: Directed Acyclic Graph of Factors Affecting the QT Interval in Malaria 12](#_Toc30471407)

[Supplementary Results 13](#_Toc30471408)

[Data Availability 13](#_Toc30471409)

[Table A: Availability of Published Datasets by Study Year of Publication 13](#_Toc30471410)

[Table B: Availability of Unpublished Datasets by Study Year of Enrolment 13](#_Toc30471411)

[Figure C: Availability of Datasets by Study Location – Asia-Pacific 14](file:///C:\Users\Xin%20Hui\Dropbox\QTc\Papers\QT%20Interval%20in%20Malaria\PLoS%20Medicine\Acceptance\S2_Appendix.docx#_Toc30471412)

[Figure D: Availability of Datasets by Study Location – Africa & Europe 15](file:///C:\Users\Xin%20Hui\Dropbox\QTc\Papers\QT%20Interval%20in%20Malaria\PLoS%20Medicine\Acceptance\S2_Appendix.docx#_Toc30471413)

[Figure E: Availability of Datasets by Study Location – Americas 16](file:///C:\Users\Xin%20Hui\Dropbox\QTc\Papers\QT%20Interval%20in%20Malaria\PLoS%20Medicine\Acceptance\S2_Appendix.docx#_Toc30471414)

[Data Description 17](#_Toc30471415)

[Table C: Characteristics of Included Studies 17](#_Toc30471416)

[Table D: Additional Characteristics of Included Population 20](#_Toc30471417)

[Table E: Comparison of Characteristics of Included and Excluded Studies 21](#_Toc30471418)

[Table F: Risk of Bias Assessment of Included and Excluded Studies 22](#_Toc30471419)

[Table G: Characteristics of Excluded Participants 30](#_Toc30471420)

[Statistical Analysis 32](#_Toc30471421)

[Table H: Model Comparison for Main Analysis of All Participants 32](#_Toc30471422)

[Sensitivity Analyses – Alternative RR Interval Transformation for Main Analysis of All Participants 33](#_Toc30471423)

[Table I: Multivariable Regression Results from Hierarchical Generalised Additive Model 33](#_Toc30471424)

[Table J: Predicted QT Intervals at Baseline and in Recovery from Malaria and Fever 33](#_Toc30471425)

[Sensitivity Analyses – Addition of TdP Risk Factor Exclusion Term for Main Analysis of All Participants 34](#_Toc30471426)

[Table K: Multivariable Regression Results from Hierarchical Generalised Additive Model 34](#_Toc30471427)

[Table L: Model Comparison 34](#_Toc30471428)

[Sensitivity Analyses – Addition of Haemoglobin Term for Main Analysis of All Participants 35](#_Toc30471429)

[Table M: Multivariable Regression Results from Hierarchical Generalised Additive Model 35](#_Toc30471430)

[Table N: Model Comparison 35](#_Toc30471431)

[Sensitivity Analyses – Addition of Parasitaemia Terms for Subgroup Analysis of Malaria Patients Only 36](#_Toc30471432)

[Table O: Multivariable Regression Results from Hierarchical Generalised Additive Model 36](#_Toc30471433)

[Table P: Model Comparison 36](#_Toc30471434)

[Sensitivity Analyses – Alternative Model Formulation for Non-Linear QT-RR Relationship for All Participants 37](#_Toc30471435)

[Table Q: Multivariable Regression Results from Hierarchical Generalised Additive Model 37](#_Toc30471436)

[References 38](#_Toc30471437)

# Supplementary Methods

## Search Strategy

An electronic literature search was conducted of the MEDLINE, EMBASE, and Global Health databases.

We searched for studies of the quinoline and structurally-related antimalarials amodiaquine, chloroquine, halofantrine, lumefantrine, mefloquine, piperaquine, primaquine, pyronaridine, and quinine for malaria-related indications in human participants with and without clinical *Plasmodium falciparum* and/or *P. vivax* malaria in which electrocardiograms (ECGs) were recorded at documented timepoints before and after drug administration.

We searched for malaria type, antimalarial drug names, and levels of repolarisation-related cardiovascular toxicity as title, abstract, and subject heading keywords, using synonyms and variant spellings as additional search terms.

We excluded animal studies, but did not apply language or publication date limits. Review articles, pooled analyses, case reports, commentary/correspondence articles, and conference abstracts were also excluded. All references were imported into EndNote bibliographic software, de-deduplicated, and screened against eligibility criteria using the Covidence software platform.

E.g. Medline search on 21 August 2017

| # ▲ | Searches |
| --- | --- |
| 1 | Malaria/ |
| 2 | Malaria, Cerebral/ |
| 3 | Malaria, Falciparum/ |
| 4 | Malaria, Vivax/ |
| 5 | plasmodium falciparum/ |
| 6 | plasmodium vivax/ |
| 7 | malaria.ti,ab. |
| 8 | falciparum.ti,ab. |
| 9 | vivax.ti,ab. |
| 10 | plasmodium.ti,ab. |
| 11 | 1 or 2 or 3 or 4 or 5 or 6 or 7 or 8 or 9 or 10 |
| 12 | piperaquine.ti,ab. |
| 13 | chloroquine.ti,ab. |
| 14 | quinine.ti,ab. |
| 15 | amodiaquine.ti,ab. |
| 16 | lumefantrine.ti,ab. |
| 17 | benflumetol.ti,ab. |
| 18 | coartem.ti,ab. |
| 19 | halofantrine.ti,ab. |
| 20 | mefloquine.ti,ab. |
| 21 | primaquine.ti,ab. |
| 22 | (pyronaridine or pyramax).ti,ab. |
| 23 | Amodiaquine/ad, ae, ct, pk, pd, po, to, tu, me, ur, bl, aa |
| 24 | Mefloquine/ad, ae, ct, pk, pd, po, to, tu, me, ur, bl, aa |
| 25 | Chloroquine/ad, ae, ct, pk, pd, po, to, tu, me, ur, bl, aa |
| 26 | Quinine/ad, ae, ct, pk, pd, po, to, tu, me, ur, bl, aa |
| 27 | Primaquine/ad, ae, ct, pk, pd, po, to, tu, me, ur, bl, aa |
| 28 | Drug Administration Schedule/ |
| 29 | 12 or 13 or 14 or 15 or 16 or 17 or 18 or 19 or 20 or 21 or 22 or 23 or 24 or 25 or 26 or 27 or 28 |
| 30 | Electrocardiography/ |
| 31 | Electrocardiography, Ambulatory/ |
| 32 | Cardiotoxicity/ |
| 33 | Arrhythmias, Cardiac/ci, co, di, pp |
| 34 | Heart Conduction System/ab, de, pp |
| 35 | Long QT Syndrome/ci |
| 36 | Torsades de Pointes/ci |
| 37 | Cardiovascular Diseases/ci, co |
| 38 | Heart/de |
| 39 | Heart rate/de, ph, pd |
| 40 | Blood Pressure/co, de, pd, ph, th |
| 41 | (QT or QTc).ti,ab. |
| 42 | (electrocardiogra$ or ECG).ti,ab. |
| 43 | cardiotoxicity.ti,ab. |
| 44 | toxic$.ti,ab. |
| 45 | safety.ti,ab. |
| 46 | (adverse adj effect$).ti,ab. |
| 47 | (blood adj pressure).ti,ab. |
| 48 | pharmacokinetic$.ti,ab. |
| 49 | 30 or 31 or 32 or 33 or 34 or 35 or 36 or 37 or 38 or 39 or 40 or 41 or 42 or 43 or 44 or 45 or 46 or 47 or 48 |
| 50 | 11 and 29 and 49 |
|  |  |

## Study-Level Data Extraction

The following information was extracted from study publications, reports, and protocols, and where necessary, requested from study investigators:

1. Study characteristics: year of publication, recruitment period, location, antimalarial treatment indication, participant inclusion and exclusion criteria, number of study participants who had ECG monitoring
2. ECG measurement methodology: centralised or study site-based, manual or automated, cardiologist or other physician reader, intermittent or continuous, any other relevant details
3. Cardiovascular adverse events: sudden cardiac death, life-threatening ventricular tachyarrhythmias (ventricular fibrillation, ventricular tachycardia, torsade de pointes), any other clinically significant arrhythmias or cardiovascular adverse events

## Individual Patient-Level Data Standardisation

This was implemented via a bespoke Application Programming Interface in Python version 3.6.3.

### ECG Intervals

Where the same ECG recording was measured by more than one set of readers, the measurements from the more specialist set^1^ of ECG readers were selected.

Measurements from triplicate ECG recordings were averaged.

Only measurements from intermittent ECG readings were used.

#### RR Interval

Heart rates in beats per minute were converted into RR intervals in milliseconds:

- RR interval = 60000/heart rate

RR intervals were then transformed with power functions:

- sqrtRR = $\sqrt{RR}$ (Bazett’s correction-like)
- cbrtRR = $\sqrt[3]{RR}$ (Fridericia’s correction-like)

#### QT/QTc interval

Where only corrected QT intervals were available, uncorrected QT intervals were calculated as follows:

- $QT = QTcB*\sqrt{RR}$ as $QTcB =\frac{QT}{\sqrt{RR}}$ (Bazett’s correction formula)
- $QT = QTcF*\sqrt[3]{RR}$ as $QTcF =\frac{QT}{\sqrt[3]{RR}}$ (Fridericia’s correction formula)

where RR intervals are in units of seconds

### Demographics

#### Age

Age was extracted as standardised to years, and otherwise calculated based on the number of years between the subject’s date of birth and the date of the start of the study.

#### Weight

Weight was extracted as standardised to kilogrammes.

### Vital Signs

#### Temperature

Oral and tympanic body temperatures were extracted as documented in the original data^2^, and converted to degrees Celsius as required. Axillary body temperatures were extracted, converted to degrees Celsius as required, then standardised by the addition of 0.5°C to original readings.

Body temperature was standardised to degrees Celsius using the following formula:

- Temperature (°C) = [Temperature (°F) – 32] / 1.8

Temperature recordings documented to be >30 minutes apart from ECG recordings were not considered to be from the same timepoint and therefore not extracted into the pooled dataset.

### Laboratory Parameters

#### Parasitaemia

The highest parasite density available for each timepoint was extracted.

Malaria parasite count measurements were standardised as parasite density per microlitre of blood according to the following formulae before being logarithmically transformed:

- Parasitaemia = (parasite count per 500 WBC / 500) * WBC count [if WBC count available]
- Parasitaemia = (parasite count per 500 WBC / 500) * 8000 [if WBC count missing]

where WBC counts are in units of mm^3^ of blood

- Parasitaemia = parasite count per 1000 RBC * 125.6 * haematocrit [if haematocrit available]
- Parasitaemia = parasite count per 1000 RBC * 125.6 * 33 [if haematocrit missing]

where haematocrit is in units of %

#### Haemoglobin

For studies in which only haematocrit was measured, haemoglobin was calculated as follows:

- Haemoglobin (g/dl) = [haematocrit (%) – 5.62] / 2.6 as
  Haematocrit (%) = 5.62 + 2.60 x haemoglobin (g/dl)^3^

## Individual Patient-Level Data Integrity Checks

Individual patient data were checked for completeness, as well as for invalid, out-of-range, or inconsistent entries. Values incompatible with what would be observed in malaria clinical trials were considered missing. Queries were raised with study investigators and resolved where possible.

## Data Analysis

### Exploratory Analyses

Pairwise relationships among collected variables were visualised using scatterplot matrices. We also summarised correlations among individual-level variables with principal component analysis biplots to identify potential redundancy (Figure S1).

### Variable Selection

Variable selection was based on directed acyclic graphs of proposed causal relationships among collected variables informed by literature review and expert consultation^4^ used to determine minimal sufficient adjustment sets for regression modelling (Figure S2).

### Model Formulation

*m1*: QT ~ $\sqrt{RR}$ + s(age:sex) + sex + (1│study)

*m2*: QT ~ $\sqrt{RR}$ + s(age:sex) + sex + temperature + (1│study)

*m3*: QT ~ $\sqrt{RR}$ + s(age:sex) + sex + temperature + indication + (1│study)

*m4*: QT ~ $\sqrt{RR}$ + s(age:sex) + sex + temperature + indication + $\sqrt{RR}$:indication + (1│study)

where s() denotes a smooth term and : denotes an interaction between variables

### Model Priors

We used weakly informative normal prior distributions summarised below:

| **Description** | **Parameter Class** | **Prior Distribution** |
| --- | --- | --- |
| Coefficients of population-level effects/predictor variables | Coefficient | Normal (0, 50) |
| Standard deviations of group-level/varying effects and splines | Standard deviation | Normal (0, 100) |
| Standard deviation of residuals | Sigma | Normal (0, 30) |

### Model Diagnostics & Posterior Predictive Checks

Posterior distributions were estimated using Markov chain Monte Carlo (MCMC) with the Hamiltonian algorithm. Convergence of the Hamiltonian algorithm was done by running four independent chains.

For each parameter:

- Trace plots were inspected for stationarity and mixing of chains
- Effective sample size (ESS) computed to be more than 10% of total sample size
- Gelman-Rubin ($\hat{R}$) convergence statistic checked to be 1 at convergence

In addition, the following Hamiltonian Monte Carlo diagnostics were checked in ShinyStan^5^ version 2.5.0:

- Tree depth information
- Energy Bayesian Fraction of Missing Information
- Divergence information

Visual posterior predictive checks were also performed.

### Model Checking and Comparison

Comparing two models on PSIS-LOO, if the absolute estimated difference in log predictive density (elpd_diff) is larger than twice the estimated standard error, this suggests one model is expected to have better predictive performance over the other. A negative elpd_diff favours the first model, while a positive elpd_diff favours the second.

*Sensitivity Analyses*

For all participants – alternative transformation for modelling the RR interval

- Alternative RR interval transformation into cube root instead of square root term:

QT ~ $\sqrt[3]{RR}$ + s(age:sex) + sex + temperature + indication + $\sqrt[3]{RR}$:indication + (1│study)

For all participants – addition of potential confounder variables

- Addition of binary variable for whether individual was enrolled in a study with one or more TdP risk factors as exclusion criteria:
  QT ~ $\sqrt{RR}$ + s(age:sex) + sex + temperature + indication + $\sqrt{RR}$:indication + TdPriskexclusion + (1│study)
- Addition of haemoglobin as a continuous variable:
  QT ~ $\sqrt{RR}$ + s(age:sex) + sex + temperature + indication + $\sqrt{RR}$:indication + haemoglobin + (1│study)

In the subgroup of malaria patients only – addition of parasitaemia as a potential confounder

- Addition of log parasitaemia as a continuous variable only:
  QT ~ $\sqrt{RR}$ + s(age:sex) + sex + temperature + indication + $\sqrt{RR}$:indication + log parasitaemia + (1│study)
- Further addition of interaction term for log parasitaemia and treatment indication:
  QT ~ $\sqrt{RR}$ + s(age:sex) + sex + temperature + indication + $\sqrt{RR}$:indication + log parasitaemia +
  log parasitaemia:indication + (1│study)

For all participants – alternative model formulation for non-linear QT-RR relationship

- Log-log linear model with base 10 logarithmic transformation of QT and RR:

logQT ~ logRR + s(age:sex) + sex + temperature + indication + logRR:indication + (1│study)

Figure A: Principal Component Analysis Biplots of Factors Affecting the QT Interval in Malaria


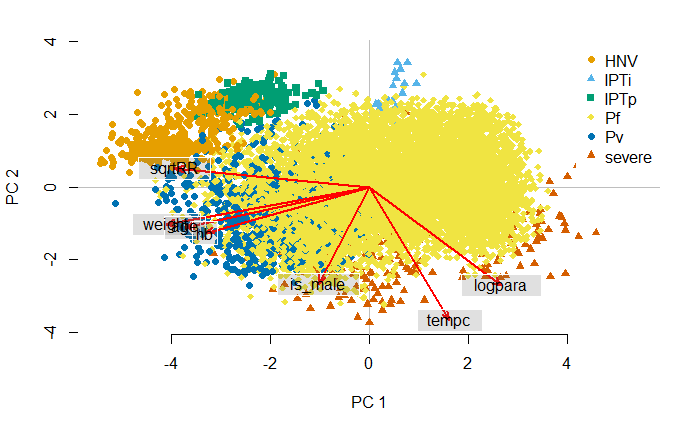


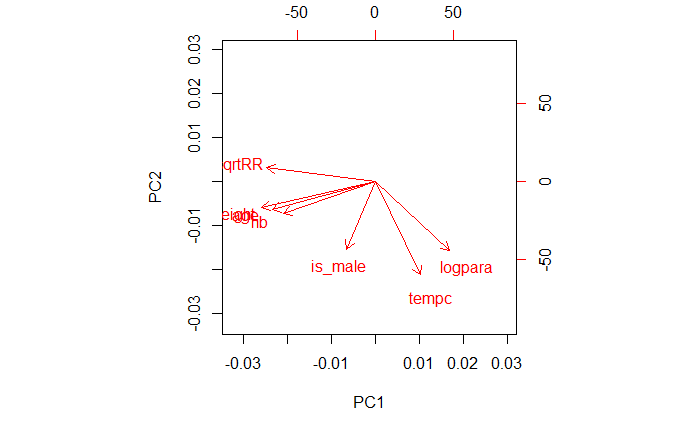


HNV = healthy volunteers, IPTi = intermittent preventive therapy in infancy, IPTp = intermittent preventive therapy in pregnancy, Pv = *P. vivax* malaria, Pf = uncomplicated *P. falciparum* malaria, severe = severe *P. falciparum* malaria
sqrtRR = $\sqrt{RR}$, is_male = sex, tempc = temperature, logpara = log(parasitaemia), hb = haemoglobin

Figure B: Directed Acyclic Graph of Factors Affecting the QT Interval in Malaria


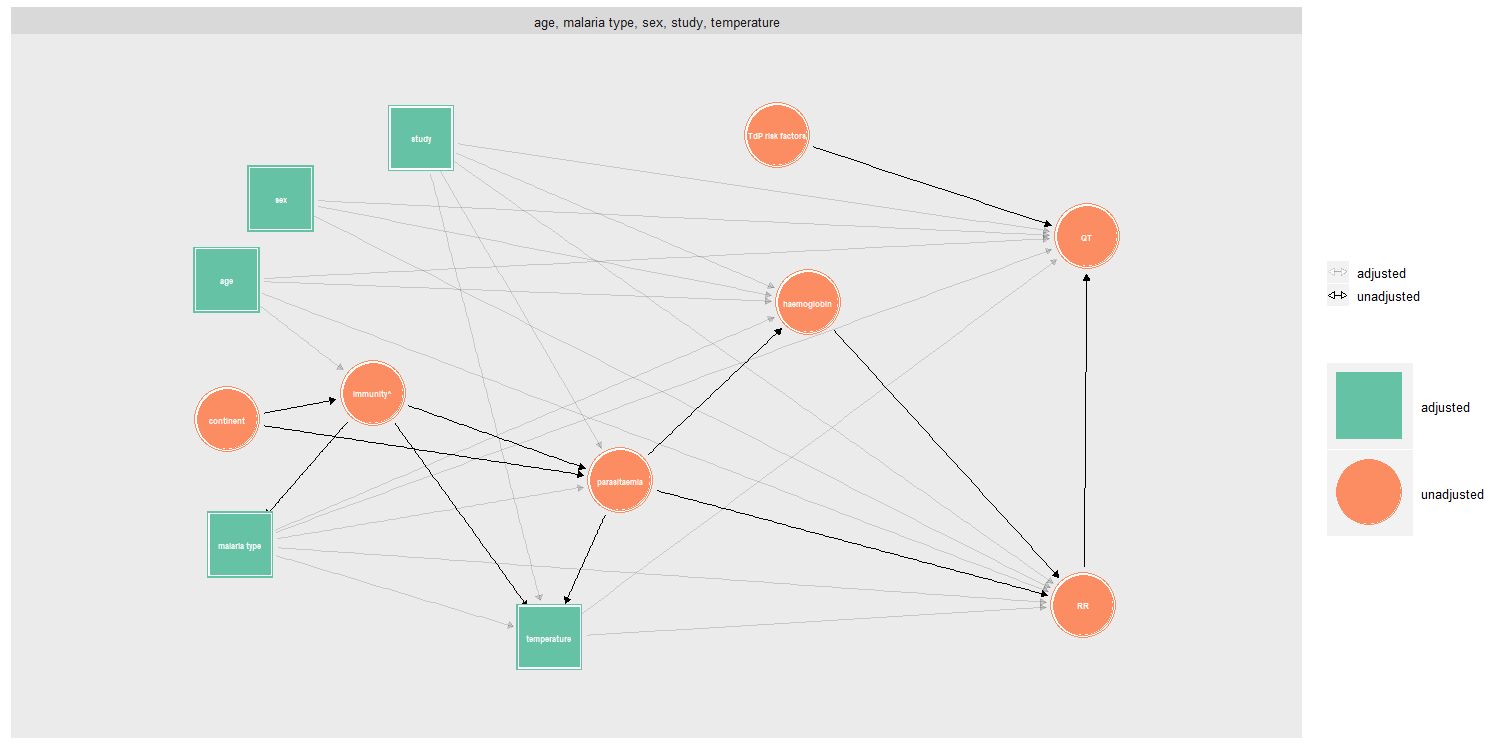


Directed acyclic graph describing proposed causal relationships among factors affecting the QT interval in malaria showing the minimal sufficient covariate adjustment set (facet label & green squares) in addition to the RR interval. The minimal adjustment set disease and demographic variables of malaria type, body temperature, age, and sex, as well as the RR interval were used as predictors and study as a varying intercept for Bayesian hierarchical multivariable regression analyses of the QT interval.

# Supplementary Results

## Data Availability

48.4% (77/159) of studies for which individual patient data were sought, and 65.1% (28/43) of included studies, were published or conducted between 2007 and 2017. 65.6% (6852/10452) of included participants were enrolled between 2007 and 2017 inclusive.

Table A: Availability of Published Datasets by Study Year of Publication

|  | ***Studies for which Data Available*** | | ***Studies for which Data Not Available*** | | |  |
| --- | --- | --- | --- | --- | --- | --- |
| **Study Year of Publication** | **Included in Meta-analysis** | **Insufficient for Inclusion** | **No Data Shared** | **No Response** | **Investigators Not Contactable** | ***Total Studies for which IPD Sought*** |
| 2012-2017 | 13 | 1 | 16 | 7 | 1 | ***38*** |
| 2007-2011 | 7 | 2 | 14 | 3 | 2 | ***28*** |
| 2002-2006 | 1 | 0 | 7 | 3 | 4 | ***15*** |
| 1997-2001 | 3 | 0 | 10 | 7 | 1 | ***21*** |
| 1992-1997 | 3 | 0 | 5 | 15 | 1 | ***24*** |
| 1988-1992 | 1 | 0 | 5 | 3 | 2 | ***11*** |
| *All Years* | *28* | *3* | *57* | *38* | *11* | ***137*** |

Table B: Availability of Unpublished Datasets by Study Year of Enrolment

|  | ***Studies for which Data Available*** | |  |
| --- | --- | --- | --- |
| **Study Year of Last Enrolment** | **Included in Meta-analysis** | **Insufficient for Inclusion** | ***Total Studies for which IPD Sought*** |
| 2012-2017 | 5 | 1 | ***6*** |
| 2007-2011 | 3 | 2 | ***5*** |
| 2002-2006 | 1 | 2 | ***3*** |
| 1997-2001 | 0 | 0 | ***0*** |
| 1992-1997 | 6 | 2 | ***8*** |
| 1988-1992 | 0 | 0 | ***0*** |
| *All Years* | *15* | *7* | ***22*** |


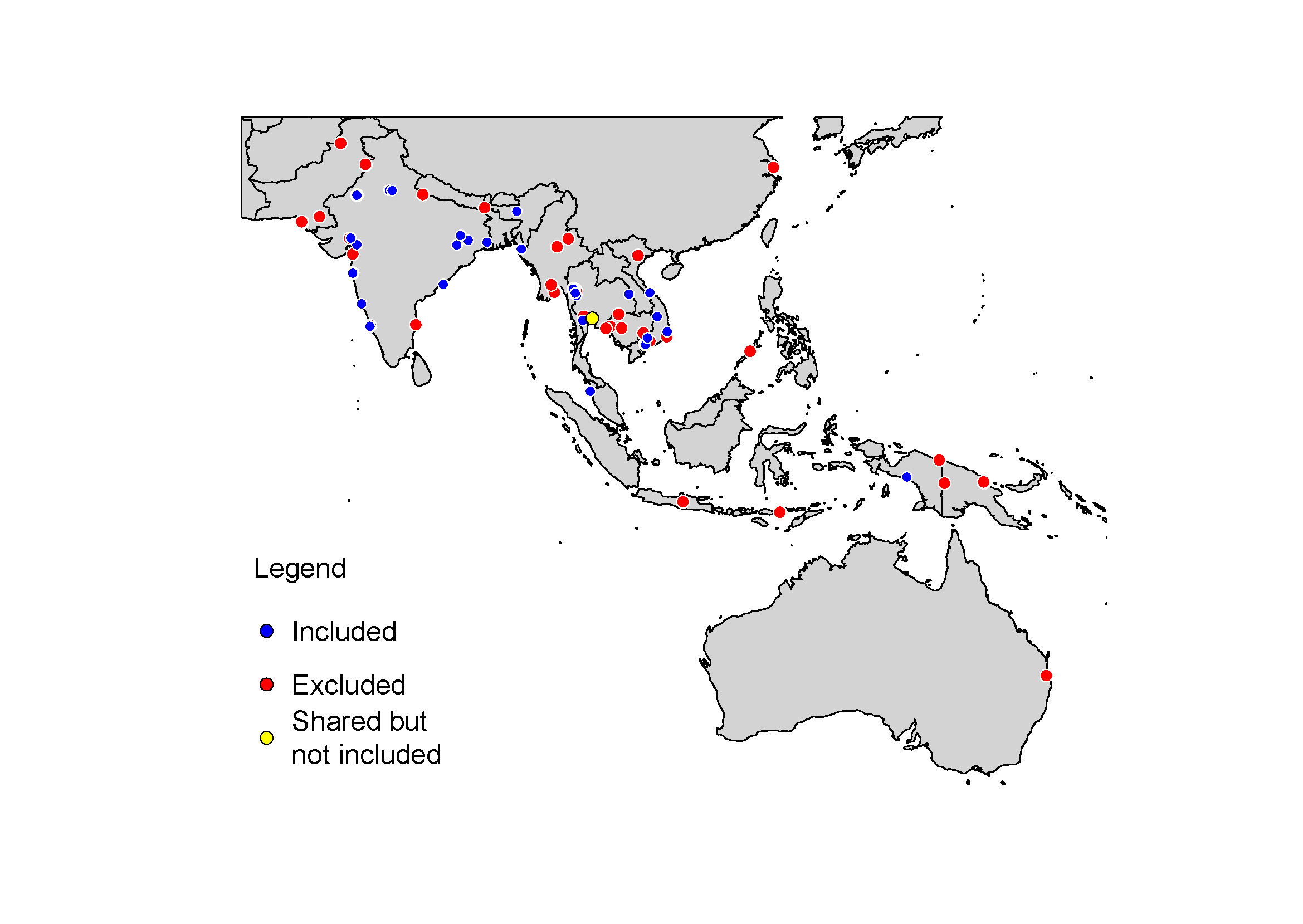


Figure C: Availability of Datasets by Study Location – Asia-Pacific

Base map from Natural Earth (www.naturalearthdata.com)

Figure D: Availability of Datasets by Study Location – Africa & Europe


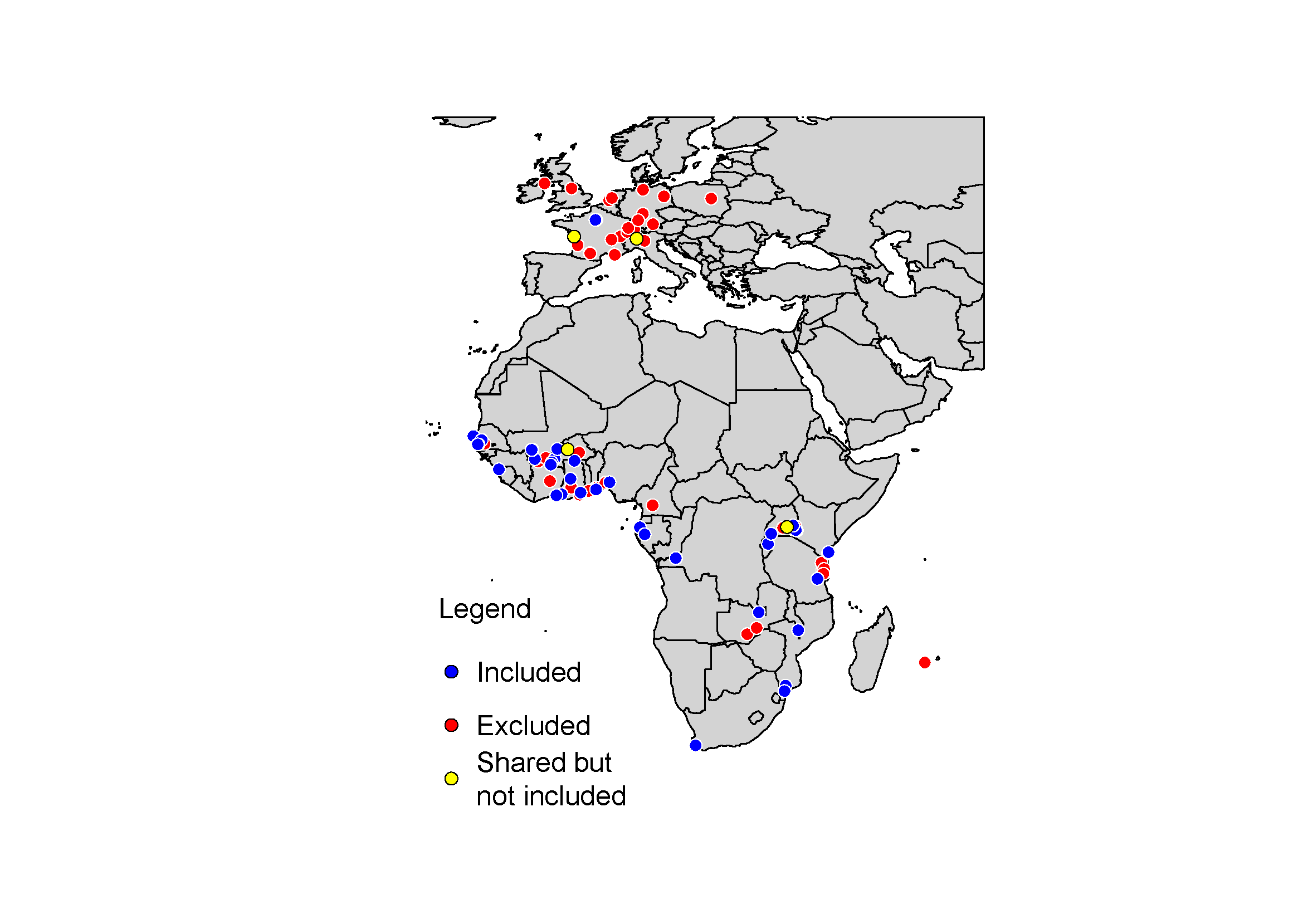


Base map from Natural Earth (www.naturalearthdata.com)


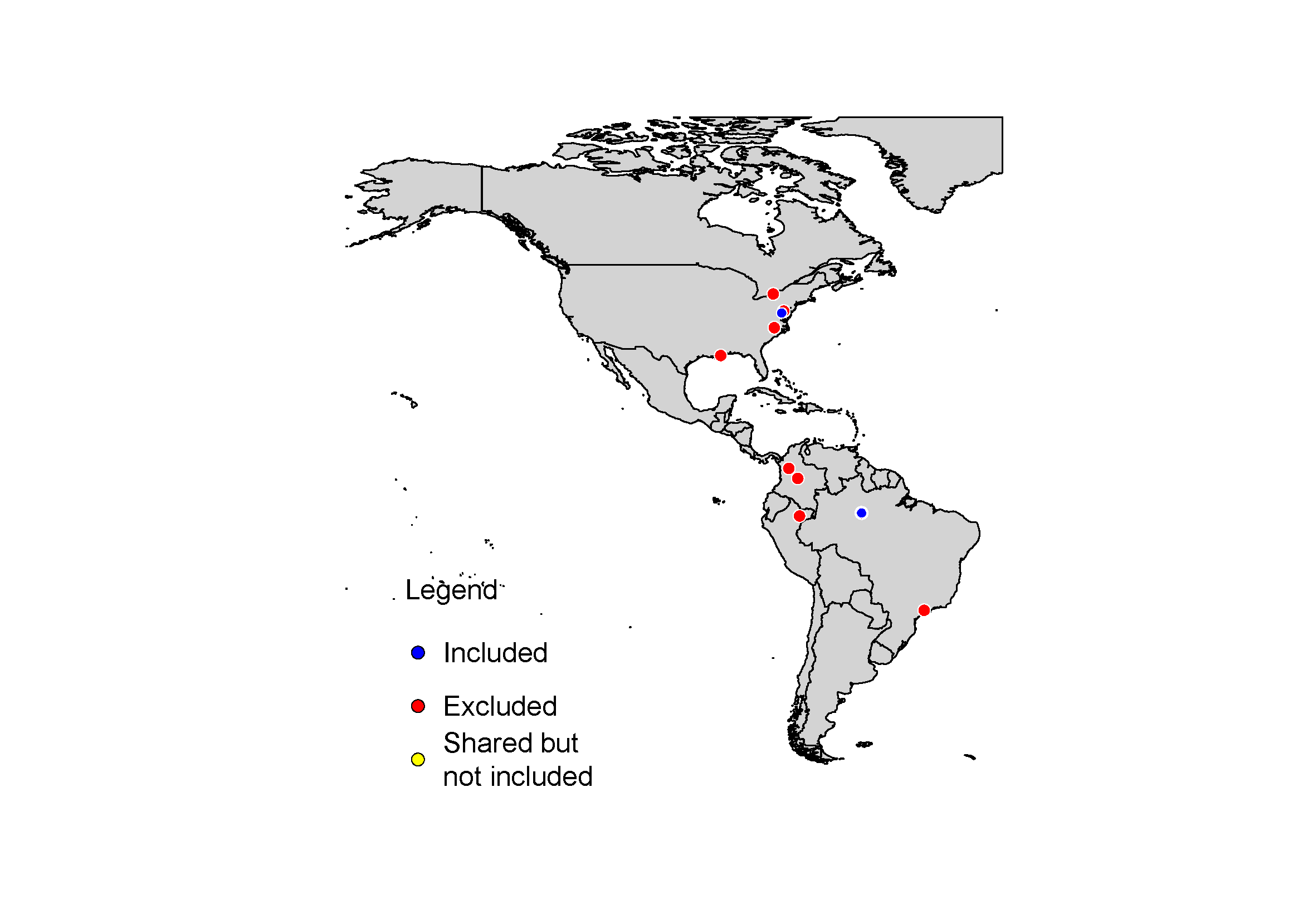


Base map from Natural Earth (www.naturalearthdata.com)

Figure E: Availability of Datasets by Study Location – Americas

## Data Description

Table C: Characteristics of Included Studies

| **Study ID** | **Country** | **Region** | **Recruitment** | **Malaria** | **Antimalarial Treatment Indication** | **Participants Enrolled** | **Participants Available** | **Participants Included** | **TdP Risk Factors Excluded** | **ECG Measurement Location** | **ECG Measurement Reader** | **ECG Measurement Method** | **Temperature Measurement Method** | **Published** |
| --- | --- | --- | --- | --- | --- | --- | --- | --- | --- | --- | --- | --- | --- | --- |
| Abernethy 2001^6^ | USA | Americas | 1995-1996 | No | Healthy volunteer pharmacokinetics | 21 | 15 | 15 | Yes | Site-based | Other physician | Intermittent | Unknown | Yes |
| Ahmed 2019^7^ | Indonesia | Asia | 2015-2016 | No | Intermittent preventive therapy - pregnancy | 33 | 33 | 28 | Yes | Centralised | Cardiologist | Intermittent | Axillary | After literature search |
| Baiden 2015^8^ | Burkina Faso, Ghana, Mozambique, Tanzania | Africa | 2013-2014 | Yes | Uncomplicated malaria - P. falciparum | 1002 | 953 | 950 | Yes | Centralised | Cardiologist | Intermittent | Axillary | Yes |
| Bassat 2009^9^ | Burkina Faso, Kenya, Mozambique, Uganda, Zambia | Africa | 2005-2006 | Yes | Uncomplicated malaria - P. falciparum | 1548 | 1536 | 1492 | No | Centralised | Cardiologist | Intermittent | Axillary | Yes |
| Bassi 2004^10^ | Nigeria | Africa | 2001 | No | Healthy volunteer pharmacokinetics | 8 | 5 | 5 | No | Site-based | Cardiologist | Intermittent | Unknown | Yes |
| Darpo 2015^11^ | Switzerland | Europe | 2012-2013 | No | Healthy volunteer pharmacokinetics | 59 | 59 | 59 | Yes | Centralised | Cardiologist | Intermittent & Continuous | Tympanic | Yes |
| Funck-Brentano 2019^12^ | France | Europe | 2010 | No | Healthy volunteer pharmacokinetics | 282 | 281 | 281 | Yes | Centralised | Cardiologist | Intermittent & Continuous | Oral | After literature search |
| Hanboonkunupakarn 2014^13^ | Thailand | Asia | 2012 | No | Healthy volunteer pharmacokinetics | 16 | 16 | 16 | Yes | Site-based | Machine | Intermittent | Axillary | Yes |
| Hanboonkunupakarn 2019^14^ | Thailand | Asia | 2014 | No | Healthy volunteer pharmacokinetics | 14 | 14 | 14 | Yes | Site-based | Machine | Intermittent | Axillary | After literature search |
| Jittamala 2011 | Thailand | Asia | 2011 | No | Healthy volunteer pharmacokinetics | 10 | 10 | 10 | Yes | Site-based | Machine | Intermittent | Axillary | No |
| Kredo 2011^15^ | South Africa | Africa | 2008-2009 | No | Healthy volunteer pharmacokinetics | 36 | 36 | 36 | Yes | Site-based | Cardiologist | Intermittent | Unknown | Yes |
| Kredo 2016^16^ | South Africa | Africa | 2009 | No | Healthy volunteer pharmacokinetics | 16 | 16 | 16 | Yes | Site-based | Cardiologist | Intermittent | Unknown | Yes |
| Krudsood 2010^17^ | Thailand | Asia | 2004-2005 | Yes | Uncomplicated malaria - P. falciparum | 50 | 50 | 50 | No | Centralised | Cardiologist | Intermittent | Axillary | Yes |
| Looareesuwan 2005 | Thailand | Asia | 2005 | Yes | Uncomplicated malaria - P. falciparum | 25 | 25 | 25 | Yes | Centralised | Cardiologist | Intermittent | Oral | No |
| Macintyre 2017^18^ | Benin, Burkina Faso, DR Congo, Gabon, Mozambique, Uganda, Vietnam | Africa & Asia | 2014-2015 | Yes | Uncomplicated malaria - P. falciparum | 437 | 440 | 435 | Yes | Centralised | Cardiologist | Intermittent | Axillary or Tympanic | After literature search |
| Mytton 2007^19^ | Thailand | Asia | 2002-2003 | Yes | Uncomplicated malaria - P. falciparum | 56 | 58 | 58 | No | Site-based | Other physician | Intermittent | Tympanic | Yes |
| Navaratnam 2009^20^ | Malaysia | Asia | 2005 | No | Healthy volunteer pharmacokinetics | 23 | 24 | 24 | Yes | Centralised | Cardiologist | Intermittent | Unknown | Yes |
| Ndiaye 2011^21^ | Senegal | Africa | 2007-2008 | Yes | Uncomplicated malaria - P. falciparum | 171 | 148 | 148 | Yes | Centralised | Cardiologist | Intermittent | Axillary | Yes |
| Nosten 1993i^22^ | Thailand | Asia | 1992 | Yes | Uncomplicated malaria - P. falciparum | 51 | 51 | 17 | No | Site-based | Other physician | Intermittent | Axillary | Yes |
| Nosten 1993ii^22^ | Thailand | Asia | 1992 | Yes | Uncomplicated malaria - P. falciparum | 10 | 9 | 7 | No | Site-based | Other physician | Intermittent | Axillary | Yes |
| Nosten 1993iii^22^ | Thailand | Asia | 1992 | Yes | Uncomplicated malaria - P. falciparum | 53 | 64 | 33 | No | Site-based | Other physician | Intermittent | Axillary | Yes |
| Ogutu 2014^23^ | Kenya | Africa | 2007-2008 | Yes | Uncomplicated malaria - P. falciparum | 54 | 51 | 51 | Yes | Centralised | Cardiologist | Intermittent | Axillary | Yes |
| Price 1995^24^ | Thailand | Asia | 1993-1994 | Yes | Uncomplicated malaria - P. falciparum | 140 | 140 | 84 | No | Site-based | Other physician | Intermittent | Axillary | Yes |
| Price 1997i^25^ | Thailand | Asia | 1994 | Yes | Uncomplicated malaria - P. falciparum | 29 | 29 | 29 | No | Site-based | Other physician | Intermittent | Axillary | Without documenting ECGs |
| Price 1997ii^25^ | Thailand | Asia | 1994-1995 | Yes | Uncomplicated malaria - P. falciparum | 13 | 13 | 13 | No | Site-based | Other physician | Intermittent | Axillary | Without documenting ECGs |
| Price 1998a^26^ | Thailand | Asia | 1994-1995 | Yes | Uncomplicated malaria - P. falciparum | 6 | 6 | 5 | No | Site-based | Other physician | Intermittent | Axillary | Without documenting ECGs |
| Price 1998b^27^ | Thailand | Asia | 1994-1995 | Yes | Uncomplicated malaria - P. falciparum | 41 | 41 | 38 | No | Site-based | Other physician | Intermittent | Axillary | Without documenting ECGs |
| PROMOTEi^28^ | Uganda | Africa | 2014-2015 | No | Intermittent preventive therapy - pregnancy | 42 | 42 | 42 | Yes | Site-based | Other physician | Intermittent | Tympanic | Yes |
| PROMOTEii^29^ | Uganda | Africa | 2014-2015 | No | Intermittent preventive therapy – pregnancy & infancy | 85 | 85 | 73 | Yes | Site-based | Other physician | Intermittent | Tympanic | After literature search |
| Pukrittayakamee 2014a^30^ | Thailand | Asia | 2010 | No | Healthy volunteer pharmacokinetics | 16 | 16 | 16 | Yes | Site-based | Machine | Intermittent | Axillary | Yes |
| Pukrittayakamee 2014b | Thailand | Asia | 2014 | No | Healthy volunteer pharmacokinetics | 15 | 15 | 15 | Yes | Site-based | Machine | Intermittent | Axillary | No |
| Siqueira 2017^31^ | Brazil | Americas | 2011-2013 | Yes | Uncomplicated malaria - P. vivax | 354 | 350 | 350 | No | Site-based | Cardiologist | Intermittent | Axillary | Yes |
| Tandon 2007 | India | Asia | 2007 | No | Healthy volunteer pharmacokinetics | 24 | 24 | 24 | Yes | Site-based | Other physician | Intermittent | Oral | No |
| Toure 2015^32^ | India,  Ivory Coast, Rwanda | Africa & Asia | 2010-2012 | Yes | Uncomplicated malaria - P. falciparum | 141 | 141 | 141 | Yes | Site-based | Other physician | Intermittent | Axillary | Yes |
| Toure 2016^33^ | Bangladesh, DR Congo, India,  Ivory Coast, Malawi, Mozambique, Senegal, Thailand | Africa & Asia | 2009-2012 | Yes | Uncomplicated malaria - P. falciparum | 1073 | 1073 | 1031 | Yes | Site-based | Other physician | Intermittent | Axillary or Oral | Yes |
| Tran 1996^34^ | Vietnam | Asia | 1992-1995 | Yes | Severe malaria | 302 | 287 | 286 | No | Site-based | Other physician | Intermittent | Axillary | Yes |
| Valecha 2010^35^ | India,  Lao PDR, Thailand | Asia | 2005-2007 | Yes | Uncomplicated malaria - P. falciparum | 1148 | 1149 | 1142 | Yes | Centralised | Cardiologist | Intermittent | Unknown | Yes |
| Valecha 2012^36^ | India, Thailand | Asia | 2007-2008 | Yes | Uncomplicated malaria - P. falciparum | 240 | 240 | 240 | Yes | Site-based | Other physician | Intermittent | Axillary or Oral | Yes |
| Valecha 2016^37^ | India | Asia | 2011-2012 | Yes | Uncomplicated malaria - P. vivax | 317 | 317 | 316 | Yes | Site-based | Other physician | Intermittent | Axillary or Oral | Yes |
| van Vugt 1999^38^ | Thailand | Asia | 1996-1997 | Yes | Uncomplicated malaria - P. falciparum | 100 | 100 | 97 | No | Site-based | Cardiologist | Intermittent | Oral | Yes |
| van Vugt 2000^39^ | Thailand | Asia | 1997-1998 | Yes | Uncomplicated malaria - P. falciparum | 199 | 199 | 198 | No | Site-based | Cardiologist | Intermittent | Oral | Yes |
| WANECAM^40,41^ | Burkina Faso, Guinea, Mali | Africa | 2011-2013 | Yes | Uncomplicated malaria - P. falciparum | 2486 | 2486 | 2485 | Yes | Centralised | Cardiologist | Intermittent | Axillary or Oral | Yes |
| White 1988^42^ | The Gambia | Africa | 1985 | Yes | Severe malaria | 62 | 65 | 57 | No | Site-based | Other physician | Intermittent | Rectal | Yes |

Table D: Additional Characteristics of Included Population

|  | **Healthy Participants**  **(n = 674)** | **Malaria Patients (n = 9778)** | **Overall  (n = 10452)** |
| --- | --- | --- | --- |
| **Weight (kg)** |  |  |  |
| Median (IQR) | 63.6 (57.0-72.2) | 33.0 (15.0-52.0) | 36.9 (15.1-54.0) |
|  |  |  |  |
| **Haemoglobin (g/dL)** |  |  |  |
| Mean (SD) | 13.5 (1.7) | 11.0 (2.3) | 11.2 (2.3) |
| <11 | 51 (7.6%) | 4856 (49.7%) | 4907 (46.9%) |
| <8 | 0 | 844 (8.6%) | 844 (8.1%) |
| <5 | 0 | 43 (0.4%) | 43 (0.4%) |
|  |  |  |  |
| **ECG Measurement Methodology** |  |  |  |
| Location of ECG interpretation |  |  |  |
| Centralised and study site-based | 392 (58.2%) | 6778 (69.3%) | 7170 (68.6%) |
| Study site-based only | 282 (41.8%) | 3000 (30.7%) | 3282 (31.4%) |
| ECG reader |  |  |  |
| Cardiologist | 449 (66.6%) | 7423 (75.9%) | 7872 (75.3%) |
| Other physician or trained personnel | 154 (22.8%) | 2355 (24.1%) | 2509 (24.0%) |
| Machine only | 71 (10.5%) | 0 | 71 (0.7%) |
|  |  |  |  |
| **Temperature Measurement Method** |  |  |  |
| Axillary | 99 (14.7%) | 7771 (79.5%) | 7870 (75.3%) |
| Oral | 305 (45.3%) | 613 (6.3%) | 918 (8.8%) |
| Tympanic | 174 (25.8%) | 195 (2.0%) | 369 (3.5%) |
| Rectal | 0 | 57 (0.6%) | 57 (0.5%) |
| Unknown | 96 (14.2%) | 1142 (11.7%) | 1238 (11.8%) |
|  |  |  |  |
| **Year of Enrolment** |  |  |  |
| 2012-2017 | 247 (36.6%) | 5316 (54.4%) | 5563 (53.2%) |
| 2007-2011 | 383 (56.8%) | 906 (9.3%) | 1289 (12.3%) |
| 1997-2006 | 29 (4.3%) | 2916 (29.8%) | 2945 (28.2%) |
| 1985-1996 | 15 (2.2%) | 621 (6.4%) | 636 (6.1%) |
| Not reported | 0 | 19 (0.2%) | 19 (0.2%) |

Table E: Comparison of Characteristics of Included and Excluded Studies

|  | **Included Studies**  **(n = 43)** | **Excluded Studies**  **(n = 116)** |
| --- | --- | --- |
| **Antimalarial Treatment Indication, studies (%)** |  |  |
| Severe/complicated malaria | 2 (4.7%) | 17 (14.7%) |
| Uncomplicated malaria | 25 (58.1%) | 61 (52.6%) |
| *P. falciparum* mono- or mixed infection | 23 (53.5%) | 52 (44.8%) |
| *P. vivax* mono-infection | 2 (4.7%) | 3 (2.6%) |
| *P. falciparum* or *P. vivax* mono- or mixed infection | 0 | 6 (5.2%) |
| Intermittent preventive therapy (IPT) | 4 (9.3%) | 8 (6.9%) |
| IPT in pregnancy (IPTp) | 3 (7.0%) | 3 (2.6%) |
| IPT in infancy (IPTi) | 1 (2.3%) | 1 (0.9%) |
| Seasonal malaria chemoprevention (SMC) | 0 | 1 (0.9%) |
| Occupational prophylaxis | 0 | 3 (2.6%) |
| Healthy volunteer pharmacokinetics | 13 (30.2%) | 30 (25.9%) |
| Healthy volunteers only | 13 (30.2%) | 27 (23.3%) |
| Healthy volunteers and uncomplicated malaria (*P. falciparum* or *P. vivax* infection) | 0 | 3 (2.6%) |
|  |  |  |
| **Geographical Region, studies (%)** |  |  |
| Asia-Pacific | 25 (58.1%) | 57 (49.1%) |
| Africa | 11 (25.6%) | 28 (24.1%) |
| Americas | 2 (4.7%) | 6 (5.2%) |
| Europe | 2 (4.7%) | 17 (14.7%) |
| Asia-Pacific & Africa | 3 (7.0%) | 4 (3.4%) |
| Others (Asia-Pacific & Americas, Africa & Europe, Americas & Europe) | 0 | 3 (2.6%) |
| Not reported | 0 | 1 (0.9%) |
|  |  |  |
| **Year Enrolment Completed, studies (%)** |  |  |
| 2007-2017 | 24 (55.8%) | 25 (21.6%) |
| Pre-2007 | 19 (44.2%) | 80 (69.0%) |
| Not reported | 0 | 11 (9.5%) |
|  |  |  |
| **Torsade de Pointes Risk Factors Excluded, studies (%)** | 26 (60.5%) | 46 (39.7%) |
|  |  |  |
| **Mean Age in Years, median (IQR)** | 26.2 (17.4-32.4) | 26.6 (16.2-31.5)^*^ |
|  |  |  |
| **Percentage of Females, median (IQR)** | 41.0 (24.4-53.2) | 28.7 (0-48.7)^†^ |
|  |  |  |
| **Risk of Bias Assessment, studies (%)** |  |  |
| Low | 39 (90.7%) | 75 (64.7%) |
| Unclear | 4 (9.3%) | 39 (33.6%) |
| High | 0 | 2 (1.7%) |

^*^Mean age not available from 8 studies ^†^Percentage not available from 14 studies

Table F: Risk of Bias Assessment of Included and Excluded Studies

|  | **Study design and objectives** | **Bias in selection of participants and constitution of study groups** | **Bias due to withdrawal or loss to follow up (attrition)** | **Information bias regarding the drug safety outcome** | **Other information bias** | **Conflict of interest** | **SUMMARY RISK OF BIAS** |
| --- | --- | --- | --- | --- | --- | --- | --- |
| *Randomised Controlled Trials - Included* | | | | | |  | |
| Abernethy 2001^6^ |  |  |  |  |  |  |  |
| Bassat 2009^9^ | 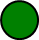 |  | 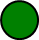 | 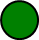 | 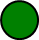 | 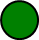 | 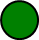 |
| Bassi 2004^10^ |  |  |  |  |  |  |  |
| Darpo 2015^11^ |  |  |  |  |  |  |  |
| Funck-Brentano 2019^12^ (subsequently published) |  |  |  |  |  |  |  |
| Hanboonkunupakarn 2014^13^ |  |  |  |  |  |  |  |
| Hanboonkunupakarn 2019^14^ (subsequently published) |  |  |  | 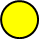 |  |  |  |
| Krudsood 2010^17^ |  |  |  |  |  |  |  |
| Macintyre 2017^18^ (subsequently published) |  |  |  |  |  |  |  |
| Mytton 2007^19^ |  |  |  |  |  |  |  |
| Navaratnam 2009^20^ |  |  |  |  |  |  |  |
| Ndiaye 2011^21^ |  |  |  |  |  |  |  |
| Ogutu 2014^23^ |  |  |  |  |  |  |  |
| Price 1995^24^ |  |  |  |  |  |  |  |
| Price 1998b^27^ |  |  |  |  |  |  |  |
| PROMOTEi^28^ |  |  |  |  |  |  |  |
| Pukrittayakamee 2014a^30^ |  |  |  |  |  |  |  |
| Pukrittayakamee 2014b (unpublished) |  |  |  |  |  |  |  |
| Siqueira 2017^31^ |  |  |  |  |  |  |  |
| Tandon 2007 (unpublished) |  |  |  |  |  |  |  |
| Toure 2015^32^ |  |  |  |  |  |  |  |
| Toure 2016^33^ |  |  |  |  |  |  |  |
| Tran 1996^34^ |  |  |  |  |  |  |  |
| Valecha 2010^35^ |  |  |  |  |  |  |  |
| Valecha 2012^36^ |  |  |  |  |  |  |  |
| Valecha 2016^37^ |  |  |  |  |  |  |  |
| van Vugt 1999^38^ |  |  |  |  |  |  |  |
| van Vugt 2000^39^ |  |  |  |  |  |  |  |
| WANECAM^40,41^ |  |  |  |  |  |  |  |
| White 1988^42^ |  |  |  |  |  |  |  |
| *Randomised Controlled Trials - Excluded* | | | | | | | |
| Abdulla 2008^43^ |  |  |  |  |  |  |  |
| Abdulla 2010^44^ |  |  |  |  |  |  |  |
| Alecrim 2006^45^ |  |  |  |  |  |  |  |
| Assimadi 2002^46^ |  |  |  |  |  |  |  |
| Benjamin 2015^47^ |  |  |  |  |  |  |  |
| Bigira 2014^48^ |  |  |  |  |  |  |  |
| Bindschedler 2000^49^ |  |  |  |  |  |  |  |
| Bindschedler 2002^50^ |  |  |  |  |  |  |  |
| Bouchaud 2000^51^ |  |  |  |  |  |  |  |
| Bouyou-Akotet 2010^52^ |  |  |  |  |  |  |  |
| Bunnag 1989^53^ |  |  |  |  |  |  |  |
| Cao 1997^54^ |  |  |  |  |  |  |  |
| D'Alessandro 2006 (unpublished) |  |  |  |  |  |  |  |
| Haroon 2005^55^ |  |  |  |  |  |  |  |
| Hien 2011^56^ |  |  |  |  |  |  |  |
| Jittamala 2015^57^ |  |  |  |  |  |  |  |
| Kakuda 2013^58^ |  |  |  |  |  |  |  |
| Karbwang 1991^59^ |  |  |  |  |  |  |  |
| Karbwang 1992a^60^ |  |  |  |  |  |  |  |
| Karbwang 1992b^61^ |  |  |  |  |  |  |  |
| Karbwang 1993b^62^ |  |  |  |  |  |  |  |
| Karbwang 1995a^63^ |  |  |  |  |  |  |  |
| Karbwang 1995b^64^ |  |  |  |  |  |  |  |
| Karbwang 1995c^65^ |  |  |  |  |  |  |  |
| Karbwang 1997^66^ |  |  |  |  |  |  |  |
| Kayentao 2012^67^ |  |  |  |  |  |  |  |
| Kervella 2006 (unpublished) |  |  |  |  |  |  |  |
| Khan 2011^68^ |  |  |  |  |  |  |  |
| Kinde-Gazard 2012^69^ |  |  |  |  |  |  |  |
| Kshirsagar 2000^70^ |  |  |  |  |  |  |  |
| Laman 2014^71^ |  |  |  |  |  |  |  |
| Lefevre 2001^72^ |  |  |  |  |  |  |  |
| Lefevre 2002a^73^ |  |  |  |  |  |  |  |
| Lefevre 2002b^74^ |  |  |  |  |  |  |  |
| Lefevre 2013^75^ |  |  |  |  |  |  |  |
| Liu 2014^76^ |  |  |  |  |  |  |  |
| Llanos-Cuentas 2014^77^ |  |  |  |  |  |  |  |
| Lon 2014^78^ |  |  |  |  |  |  |  |
| Manning 2014^79^ |  |  |  |  |  |  |  |
| Massougbodji 2002^80^ |  |  |  |  |  |  |  |
| McGready 2008^81^ | 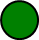 | 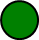 | 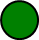 |  | 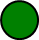 |  |  |
| Miller 2013^82^ |  |  |  | 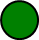 |  |  |  |
| Moore 2014^83^ |  |  |  |  |  |  |  |
| Morris 2012^84^ |  |  |  |  |  |  |  |
| Murphy 1996^85^ |  |  |  |  |  |  |  |
| Mutabingwa 2009^86^ |  |  |  |  |  |  |  |
| Mzayek 2007^87^ |  |  |  |  |  |  |  |
| Na-Bangchang 2000^88^ |  |  |  |  |  |  |  |
| Na-Bangchang 2005^89^ |  |  |  |  |  |  |  |
| Nasveld 2010^90^ |  |  |  |  |  |  |  |
| Nelwan 2015^91^ |  |  |  |  |  |  |  |
| Newton 2001^92^ |  |  |  |  |  |  |  |
| Ngouesse 2001^93^ |  |  |  |  |  |  |  |
| Nosten 1990^94^ |  |  |  |  |  |  |  |
| Nosten 1994^95^ |  |  |  |  |  |  |  |
| Olliaro 2010^96^ |  |  |  |  |  |  |  |
| Omoruyi 2007^97^ |  |  |  |  |  |  |  |
| Orrell 2008^98^ |  |  |  |  |  |  |  |
| Piola 2010^99^ |  |  |  |  |  |  |  |
| Poravuth 2011^100^ |  |  |  |  |  |  |  |
| Pyar 2007^101^ |  |  |  |  |  |  |  |
| Pyar 2009^102^ |  |  |  |  |  |  |  |
| Restrepo 1996^103^ |  |  |  |  |  |  |  |
| Rueangweerayut 2012^104^ |  |  |  |  |  |  |  |
| Sabchareon 1988^105^ |  |  |  |  |  |  |  |
| SB 1993 (unpublished) |  |  |  |  |  |  |  |
| Song 2011^106^ |  |  |  |  |  |  |  |
| Sowunmi 1990^107^ |  |  |  |  |  |  |  |
| Staedke 2018^108^ (subsequently published) |  |  |  |  |  |  |  |
| Supan 2017^109^ |  |  |  |  |  |  |  |
| Taylor 1998^110^ |  |  |  |  |  |  |  |
| Thapa 2007^111^ |  |  |  |  |  |  |  |
| Thuma 2000^112^ |  |  |  |  |  |  |  |
| Tjitra 2012^113^ |  |  |  |  |  |  |  |
| Touze 2002^114^ |  |  |  |  |  |  |  |
| Trung 2009^115^ |  |  |  |  |  |  |  |
| Tshefu 2010^116^ |  |  |  |  |  |  |  |
| van Agtmael 1999^117^ |  |  |  |  |  |  |  |
| van Hensbroek 1996^118^ |  |  |  |  |  |  |  |
| Walker 1993^119^ |  |  |  |  |  |  |  |
| *Cohorts - Included* | | | | | | | |
| Ahmed 2019^7^ (subsequently published) |  |  |  |  |  |  |  |
| Baiden 2015^8^ |  |  |  |  |  |  |  |
| Jittamala 2011 (unpublished) |  |  |  |  |  |  |  |
| Kredo 2011^15^ |  |  |  |  |  |  |  |
| Kredo 2016^16^ |  |  |  |  |  |  |  |
| Looareesuwan 2005 (unpublished) |  |  |  |  |  |  |  |
| Nosten 1993i^22^ |  |  |  |  |  |  |  |
| Nosten 1993ii^22^ |  |  |  |  |  |  |  |
| Nosten 1993iii^22^ |  |  |  |  |  |  |  |
| Price 1997i^25^ |  |  |  |  |  |  |  |
| Price 1997ii^25^ |  |  |  |  |  |  |  |
| Price 1998a^26^ |  |  |  |  |  |  |  |
| PROMOTEii^29^ (subsequently published) |  |  |  |  |  |  |  |
| *Cohorts - Excluded* | | | | | | | |
| Adjei 2012^120^ |  |  |  |  |  |  |  |
| Auprayoon 1995^121^ |  |  |  |  |  |  |  |
| Bhatt 2006^122^ |  |  |  |  |  |  |  |
| Byakika-Kibwika 2011^123^ |  |  |  |  |  |  |  |
| Claessen 1998^124^ |  |  |  |  |  |  |  |
| Davis 1988^125^ |  |  |  |  |  |  |  |
| Davis 1990^126^ |  |  |  |  |  |  |  |
| Edwards 1988^127^ |  |  |  |  |  |  |  |
| Falade 2005^128^ |  |  |  |  |  |  |  |
| Haider 2013^129^ |  |  |  |  |  |  |  |
| Hatz 2008^130^ |  |  |  |  |  |  |  |
| Hombhanje 1998^131^ |  |  |  |  |  |  |  |
| Jaspers 1996^132^ |  |  |  |  |  |  |  |
| Karbwang 1993a^133^ |  |  |  |  |  |  |  |
| Karunajeewa 2004^134^ |  |  |  |  |  |  |  |
| Khan 2006^135^ |  |  |  |  |  |  |  |
| Krishna 1993^136^ |  |  |  |  |  |  |  |
| Lavallee 2001^137^ |  |  | 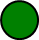 |  |  |  |  |
| Mansor 1990^138^ |  |  |  |  |  |  |  |
| Matson 1996^139^ |  |  | 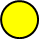 |  |  |  |  |
| Minodier 2005^140^ |  |  | 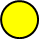 |  |  |  |  |
| Monlun 1995^141^ |  |  |  |  |  |  |  |
| Mra 1991^142^ |  |  |  |  |  |  |  |
| Na-Bangchang 1994^143^ |  |  |  |  |  |  |  |
| Nyunt 2012^144^ |  |  |  |  |  |  |  |
| Ogunkunle 2011^145^ |  |  |  |  |  |  |  |
| Roggelin 2014^146^ |  |  | 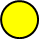 |  |  |  |  |
| Rusca 2007 (unpublished) |  |  |  |  |  |  |  |
| SB 1994 (unpublished) |  |  |  |  |  |  |  |
| Sowunmi 1998^147^ |  |  | 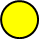 |  |  |  |  |
| Stein 2015^148^ |  |  |  |  |  |  |  |
| Sukontason 1996^149^ |  |  |  |  |  |  |  |
| Supanaranond 1997^150^ |  |  |  |  |  |  |  |
| Touze 1996^151^ |  |  |  |  |  |  |  |
| von Seidlein 1997^152^ |  |  |  |  |  |  |  |
| Win 1992^153^ |  |  |  |  |  |  |  |

**Legend**

|  | Low |  | Unclear |  | High |
| --- | --- | --- | --- | --- | --- |

As this systematic review was conducted to identify studies for an individual patient data meta-analysis, risk of bias assessment of statistical methods of individual studies was considered not relevant.

Table G: Characteristics of Excluded Participants

|  | **Healthy Participants**  **(n = 17)** | **Malaria Patients (n = 243)** | **Overall  (n = 260)** |
| --- | --- | --- | --- |
| **Antimalarial Treatment Indication** |  |  |  |
| Severe/complicated malaria |  | 9 (3.7%) | 9 (3.5%) |
| Uncomplicated malaria |  | 234 (96.3%) | 234 (90%) |
| *P. falciparum* mono- or mixed infection |  | 233 (95.9%) | 233 (89.6%) |
| *P. vivax* mono-infection |  | 1 (0.4%) | 1 (0.38%) |
| Intermittent preventive therapy (IPT) | 17 (100%) |  | 17 (6.5%) |
| Pregnancy (IPTp) | 5 (29.4%) |  | 5 (1.9%) |
| Infancy (IPTi) | 12 (70.6%) |  | 12 (4.6%) |
| Healthy volunteer pharmacokinetics | 0 |  | 0 |
|  |  |  |  |
| **Age (years)** |  |  |  |
| Median (IQR) | 0.61 (0.61-0.62)^*^ | 14.0 (5.0-24.0) | 13.0 (4.0-24.0)^*^ |
| <15 | 12 (70.6%) | 128 (52.7%) | 140 (54.1%) |
| <1 | 12 (70.6%) | 4 (1.6%) | 16 (6.2%) |
| 1-<5 | 0 | 51 (21.0%) | 51 (19.7%) |
| 5-<15 | 0 | 73 (30.0%) | 73 (28.2%) |
| ≥15 | 4 (23.5%) | 115 (47.3%) | 119 (45.9%) |
| ≥35 | 1 (5.9%) | 39 (16.0%) | 40 (15.4%) |
| ≥50 | 0 | 13 (5.3%) | 13 (5.0%) |
|  |  |  |  |
| **Weight (kg)** |  |  |  |
| Median (IQR) | 7.8 (7.6-8.6) | 37.6 (15.0-49.7)^†^ | 37.0 (13.8-49.1)^†^ |
|  |  |  |  |
| **Sex** |  |  |  |
| Female | 11 (64.7%) | 112 (46.1%) | 123 (47.3%) |
| Pregnant | 5 (29.4%) | 0 | 5 (1.9%) |
| Male | 6 (35.3%) | 131 (53.9%) | 137 (52.7%) |
|  |  |  |  |
| **Temperature (°C)** |  |  |  |
| Mean (SD) | 37.1 (0.4) | 38.1 (1.3)^‡^ | 38.0 (1.3)^‡^ |
| ≥37.5 | 1 (5.9%) | 74 (61.7%) | 75 (54.7%) |
|  |  |  |  |
| **Parasitaemia (parasites/μL)** |  |  |  |
| Median (IQR) | N/A | 8327 (1306-30142)^§^ | 8327 (1306-30142)^§^ |
| ≥10,000 | N/A | 105 (47.3%) | 105 (43.9%) |
| ≥50,000 | N/A | 38 (17.1%) | 38 (15.9%) |
| ≥100,000 | N/A | 19 (8.6%) | 19 (7.9%) |
| ≥250,000 | N/A | 2 (0.9%) | 2 (0.84%) |
|  |  |  |  |
| **Haemoglobin (g/dL)** |  |  |  |
| Mean (SD) | 10.4 (1.3)^\|^ | 11.7 (2.1)^¶^ | 11.7 (2.1)^**^ |
| <11 | 2 (66.7%) | 38 (30.4%) | 40 (31.3%) |
| <8 | 0 | 5 (4.0%) | 5 (3.9%) |
| <5 | 0 | 0 | 0 |
|  |  |  |  |
| **Heart Rate (beats per minute)** |  |  |  |
| Mean (SD) | 122 (18) | 107 (31) | 108 (31) |
| ≥140 | 0 | 40 (16.5%) | 40 (15.4%) |
| 120-139 | 12 (70.6%) | 29 (11.9%) | 41 (15.8%) |
| 100-119 | 2 (11.8%) | 61 (25.1%) | 63 (24.2%) |
| 80-99 | 3 (17.6%) | 62 (25.5%) | 65 (25.0%) |
| 60-79 | 0 | 44 (18.1%) | 44 (16.9%) |
| <60 | 0 | 7 (2.9%) | 7 (2.7%) |
|  |  |  |  |
| **Torsade de Pointes Risk Factors** |  |  |  |
| Excluded from the individual study | 17 (100%) | 59 (24.3%) | 76 (29.2%) |
| Not excluded from the individual study | 0 | 184 (75.7%) | 184 (70.8%) |
|  |  |  |  |
| **Geographical Region** |  |  |  |
| Africa | 12 (70.6%) | 102 (42.0%) | 114 (43.8%) |
| Asia | 5 (29.4%) | 141 (58.0%) | 146 (56.2%) |
|  |  |  |  |
| **Year of Enrolment** |  |  |  |
| 2012-2017 | 17 (100%) | 51 (21.0%) | 68 (26.2%) |
| 2007-2011 | 0 | 2 (0.82%) | 2 (0.77%) |
| 1997-2006 | 0 | 51 (21.0%) | 51 (19.6%) |
| 1985-1996 | 0 | 139 (57.2%) | 139 (53.5%) |

^*^1 participant had missing age; ^†^1 participant had missing weight; ^‡^123 participants had missing temperature; ^§^21 participants had missing parasitaemia; ^|^14 participants had missing haemoglobin; ^¶^118 participants had missing haemoglobin; ^**^132 participants had missing haemoglobin

## Statistical Analysis

Table H: Model Comparison for Main Analysis of All Participants

For model formulations, please see *Supplementary Methods – Data Analysis* on page 8 of this appendix.

| **Models Compared** | **Difference in Estimated Log Predictive Density (elpd_diff)** | **Standard Error (SE)** | **Interpretation** |
| --- | --- | --- | --- |
| *m1* – *m2* | 240.34 | 32.28 | Favours m2 |
| *m1* – *m3* | 240.43 | 32.29 | Favours m3 |
| *m1* – *m4* | 371.95 | 47.10 | Favours m4 |
| *m2* – *m3* | 0.09 | 0.89 | Does not favour m2 or m3 |
| *m2* – *m4* | 131.60 | 33.54 | Favours m4 |
| *m3* – *m4* | 131.52 | 33.54 | Favours m4 |

Model expected predictive performance was improved by addition of temperature (*m2*) as well as malaria type both as an independent term and as an interaction term with $\sqrt{RR}$ (*m4*) but not when malaria type was added as an independent term alone (*m3*). Overall, *m4* was the best model.

### Sensitivity Analyses – Alternative RR Interval Transformation for Main Analysis of All Participants

Table I: Multivariable Regression Results from Hierarchical Generalised Additive Model

| **Predictor** | **Number of Participants** | **Estimate (95% Credible Interval) /  Smooth Description** | **Clinically Significant?** |
| --- | --- | --- | --- |
| $\sqrt[3]{RR}$ interval, per ^3^√millisecond increase (healthy participants) | 10452 | 43.36 (40.82, 45.81) milliseconds | Yes |
| $\sqrt[3]{RR}$ interval, per ^3^√millisecond increase  (by malaria type vs healthy participants) | 10452 |  | Yes |
| Healthy participants | 674 | Reference |  |
| Uncomplicated vivax malaria | 666 | 1.00 (-2.09, 4.10) milliseconds |  |
| Uncomplicated falciparum malaria | 8769 | 7.11 (4.57, 9.73) milliseconds |  |
| Severe/complicated malaria | 343 | 16.87 (12.62, 21.16) milliseconds |  |
| Age | 10452 |  | Yes |
| Female | 4252 | Lengthens by ~8 milliseconds over childhood, then lengthens more gradually by another  ~5 milliseconds in adulthood |  |
| Male | 6200 | Lengthens by ~8 milliseconds over childhood, then shortens by ~10 milliseconds around puberty before gradually lengthening by  ~10 milliseconds in adulthood |  |
| Sex | 10452 |  | Yes |
| Female | 4252 | Reference |  |
| Male | 6200 | -4.23 (-4.99, -3.46) milliseconds |  |
| Body temperature, per 1°C increase | 10452 | -2.67 (-3.04, -2.30) milliseconds | Yes |
| Malaria Type | 10452 |  | Yes |
| Healthy participants | 674 | Reference |  |
| Uncomplicated vivax malaria | 666 | -3.08 (-35.47, 29.22) milliseconds |  |
| Uncomplicated falciparum malaria | 8769 | -64.42 (-90.31, -39.01) milliseconds |  |
| Severe/complicated malaria | 343 | -130.84 (-169.47, -91.96) milliseconds |  |

Table J: Predicted QT Intervals at Baseline and in Recovery from Malaria and Fever

|  | **Healthy** | **Uncomplicated  vivax** | **Uncomplicated  falciparum** | **Severe malaria** |
| --- | --- | --- | --- | --- |
| QT interval at baseline, milliseconds (95% PI)  [HR=100bpm] | 327 (283-395)  [T=36.5°C] | 328 (281-371)  [T=38.5°C] | 318 (275-358)  [T=38.5°C] | 333 (288-377)  [T=38.5°C] |
| QT interval in recovery, milliseconds (95% PI)  [HR=60bpm] | 396 (352-436)  [T=36.5°C] | 402 (356-446) [T=36.5°C] | 403 (359-443) [T=36.5°C] | 433 (386-477) [T=36.5°C] |
| QT lengthening from baseline, milliseconds | 69 | 74 | 85 | 100 |
| Additional QT lengthening from baseline compared to healthy subject, milliseconds | 0 | 5 | 16 | 31 |
| Malaria-related QT lengthening from baseline, % | 0 | 7 | 19 | 31 |

PI = prediction interval, HR = heart rate, bpm = beats per minute, T = body temperature

Predicted values for a 25-year-old male from multivariable hierarchical generalised additive model adjusting for heart rate/RR interval (as $\sqrt[3]{RR}$), age, sex, malaria type, body temperature, and individual study effects

### Sensitivity Analyses – Addition of TdP Risk Factor Exclusion Term for Main Analysis of All Participants

Table K: Multivariable Regression Results from Hierarchical Generalised Additive Model

| **Predictor** | **Number of Participants** | **Estimate (95% Credible Interval) /  Smooth Description** | **Clinically Significant?** |
| --- | --- | --- | --- |
| $\sqrt{RR}$interval, per √millisecond increase (healthy participants) | 10452 | 9.17 (8.60, 9.74) milliseconds | Yes |
| $\sqrt{RR}$ interval, per √millisecond increase  (by malaria type vs healthy participants) | 10452 |  | Yes |
| Healthy participants | 674 | Reference |  |
| Uncomplicated vivax malaria | 666 | 0.62 (-0.09, 1.34) milliseconds |  |
| Uncomplicated falciparum malaria | 8769 | 2.24 (1.66, 2.82) milliseconds |  |
| Severe/complicated malaria | 343 | 4.88 (3.89, 5.91) milliseconds |  |
| Age | 10452 |  | Yes |
| Female | 4252 | Lengthens by ~8 milliseconds over childhood, then lengthens more gradually by another  ~5 milliseconds in adulthood |  |
| Male | 6200 | Lengthens by ~8 milliseconds over childhood, then shortens by ~10 milliseconds around puberty before gradually lengthening by  ~10 milliseconds in adulthood |  |
| Sex | 10452 |  | Yes |
| Female | 3909 | Reference |  |
| Male | 5869 | -4.23 (-5.00, -3.45) milliseconds |  |
| Body temperature, per 1°C increase | 10452 | -2.80 (-3.16, -2.43) milliseconds | Yes |
| Malaria Type | 10452 |  | Yes |
| Healthy participants | 674 | Reference |  |
| Uncomplicated vivax malaria | 666 | -11.15 (-37.34, 15.24) milliseconds |  |
| Uncomplicated falciparum malaria | 8769 | -61.25 (-80.23, -42.60) milliseconds |  |
| Severe/complicated malaria | 343 | -109.89 (-140.03, -78.95) milliseconds |  |
| Torsade de Pointes Risk Factors Excluded from Individual Study | 10452 |  | No |
| No | 2819 | Reference |  |
| Yes | 7633 | -0.78 (-9.69, 7.88) milliseconds |  |

Table L: Model Comparison

For model formulations, please see *Supplementary Methods – Data Analysis* on pages 8 of this appendix.

| **Models Compared** | **Difference in Estimated Log Predictive Density (elpd_diff)** | **Standard Error (SE)** | **Interpretation** |
| --- | --- | --- | --- |
| *m4* – (*m4 + TdPriskexclusion)* | -0.97 | 0.32 | Favours m4 |

Model expected predictive performance was not improved by addition of the TdP risk factor exclusion term.

### Sensitivity Analyses – Addition of Haemoglobin Term for Main Analysis of All Participants

Table M: Multivariable Regression Results from Hierarchical Generalised Additive Model

| **Predictor** | **Number of Participants** | **Estimate (95% Credible Interval) /  Smooth Description** | **Clinically Significant?** |
| --- | --- | --- | --- |
| $\sqrt{RR}$ interval, per √millisecond increase (healthy participants) | 10452 | 9.16 (8.59, 9.71) milliseconds | Yes |
| $\sqrt{RR}$ interval, per √millisecond increase  (by malaria type vs healthy participants) | 10452 |  | Yes |
| Healthy participants | 674 | Reference |  |
| Uncomplicated vivax malaria | 666 | 0.65 (-0.05, 1.38) milliseconds |  |
| Uncomplicated falciparum malaria | 8769 | 2.25 (1.68, 2.83) milliseconds |  |
| Severe/complicated malaria | 343 | 4.81 (3.81, 5.84) milliseconds |  |
| Age | 10452 |  | Yes |
| Female | 4252 | Lengthens by ~8 milliseconds over childhood, then lengthens more gradually by another  ~5 milliseconds in adulthood |  |
| Male | 6200 | Lengthens by ~8 milliseconds over childhood, then shortens by ~10 milliseconds around puberty before gradually lengthening by  ~10 milliseconds in adulthood |  |
| Sex | 10452 |  | Yes |
| Female | 3909 | Reference |  |
| Male | 5869 | -3.87 (-4.66, -3.07) milliseconds |  |
| Body temperature, per 1°C increase | 10452 | -2.76 (-3.13, -2.38) milliseconds | Yes |
| Malaria Type | 10452 |  | Yes |
| Healthy participants | 674 | Reference |  |
| Uncomplicated vivax malaria | 666 | -12.67 (-38.78, 12.57) milliseconds |  |
| Uncomplicated falciparum malaria | 8769 | -62.63 (-81.47, -44.08) milliseconds |  |
| Severe/complicated malaria | 343 | -110.75 (-139.99, -81.57) milliseconds |  |
| Haemoglobin, per g/dL increase | 10452 | -0.51 (-0.72, -0.30) milliseconds | No |

Table N: Model Comparison

For model formulations, please see *Supplementary Methods – Data Analysis* on pages 8 of this appendix.

| **Models Compared** | **Difference in Estimated Log Predictive Density (elpd_diff)** | **Standard Error (SE)** | **Interpretation** |
| --- | --- | --- | --- |
| *m4* – (*m4 + haemoglobin)* | 19.89 | 10.65 | Does not favour either model |

Model expected predictive performance was not improved by addition of the haemoglobin term.

### Sensitivity Analyses – Addition of Parasitaemia Terms for Subgroup Analysis of Malaria Patients Only

Table O: Multivariable Regression Results from Hierarchical Generalised Additive Model

| **Predictor** | **Number of Participants** | **Estimate (95% Credible Interval) /  Smooth Description** | **Clinically Significant?** |
| --- | --- | --- | --- |
| $\sqrt{RR}$ interval, per √millisecond increase (uncomplicated falciparum malaria) | 9778 | 11.48 (11.29, 11.67) milliseconds | Yes |
| $\sqrt{RR}$ interval, per √millisecond increase  (by malaria type vs uncomplicated falciparum malaria) | 9778 |  | Yes |
| Uncomplicated vivax malaria | 666 | -1.68 (-2.15, -1.21) milliseconds |  |
| Uncomplicated falciparum malaria | 8769 | Reference |  |
| Severe/complicated malaria | 343 | 2.49 (1.55, 3.40) milliseconds |  |
| Age | 9778 |  | Yes |
| Female | 3909 | Lengthens by ~8 milliseconds in childhood, then lengthens more gradually by another  ~5 milliseconds in adulthood |  |
| Male | 5869 | Lengthens by ~8 milliseconds over childhood, then shortens by ~10 milliseconds around puberty before gradually lengthening by  ~10 milliseconds in adulthood |  |
| Sex | 9778 |  | Yes |
| Female | 3909 | Reference |  |
| Male | 5869 | -3.76 (-4.55, -2.95) milliseconds |  |
| Body temperature, per 1°C increase | 9778 | -2.85 (-3.22, -2.47) milliseconds | Yes |
| Malaria Type | 9778 |  | Yes |
| Uncomplicated vivax malaria | 666 | 44.83 (26.33, 63.03) milliseconds |  |
| Uncomplicated falciparum malaria | 8769 | Reference |  |
| Severe/complicated malaria | 343 | -37.56 (-66.03, -9.28) milliseconds |  |
| Parasitaemia, per 10-fold increase (uncomplicated falciparum malaria) | 9778 | 0.65 (0.17, 1.14) milliseconds | No |
| Parasitaemia, per 10-fold increase (by malaria type vs uncomplicated falciparum malaria) | 9778 |  | No |
| Uncomplicated vivax malaria | 666 | 2.10 (-0.66, 4.80) |  |
| Uncomplicated falciparum malaria | 8769 | Reference |  |
| Severe/complicated malaria | 343 | -1.90 (-3.98, 0.14) |  |

Table P: Model Comparison

For model formulations, please see *Supplementary Methods – Data Analysis* on pages 8 of this appendix.

| **Models Compared** | **Difference in Estimated Log Predictive Density (elpd_diff)** | **Standard Error (SE)** | **Interpretation** |
| --- | --- | --- | --- |
| *m4* – (*m4 + logpara)* | 5.58 | 5.35 | Does not favour either model |
| *m4 – (m4 + logpara by indication)* | 3.73 | 8.45 | Does not favour either model |
| *(m4 + logpara) – (m4 + logpara by indication)* | -1.85 | 8.03 | Does not favour either model |

Model expected predictive performance was not improved by addition of parasitaemia terms.

### Sensitivity Analyses – Alternative Model Formulation for Non-Linear QT-RR Relationship for All Participants

Table Q: Multivariable Regression Results from Hierarchical Generalised Additive Model

| **Predictor** | **Number of Participants** | **Estimate (95% Credible Interval) /  Smooth Description** | **Clinically Significant?** |
| --- | --- | --- | --- |
| logRR interval, per log(millisecond) increase (healthy participants) | 10452 | 0.36 (0.34, 0.39) log(milliseconds) | Yes |
| logRR interval, per log(millisecond) increase  (by malaria type vs healthy participants) | 10452 |  | Yes |
| Healthy participants | 674 | Reference |  |
| Uncomplicated vivax malaria | 666 | 0.014 (-0.018, 0.047) log(milliseconds) |  |
| Uncomplicated falciparum malaria | 8769 | 0.083 (0.055, 0.11) log(milliseconds) |  |
| Severe/complicated malaria | 343 | 0.20 (0.15, 0.24) log(milliseconds) |  |
| Age | 10452 |  | Yes |
| Female | 4252 | Lengthens over childhood, then lengthens more gradually in adulthood |  |
| Male | 6200 | Lengthens over childhood, then shortens by around puberty before gradually lengthening in adulthood |  |
| Sex | 10452 |  | Yes |
| Female | 4252 | Reference |  |
| Male | 6200 | -0.0055 (-0.0065, -0.0044) log(milliseconds) |  |
| Body temperature, per 1°C increase | 10452 | -0.0037 (-0.0042, -0.0032) log(milliseconds) | Yes |
| Malaria Type | 10452 |  | Yes |
| Healthy participants | 674 | Reference |  |
| Uncomplicated vivax malaria | 666 | -0.034 (-0.13, 0.064) log(milliseconds) |  |
| Uncomplicated falciparum malaria | 8769 | -0.24 (-0.32, -0.15) log(milliseconds) |  |
| Severe/complicated malaria | 343 | -0.54 (-0.66, -0.41) log(milliseconds) |  |

# References

1. Viskin S, Rosovski U, Sands AJ, et al. Inaccurate electrocardiographic interpretation of long QT: the majority of physicians cannot recognize a long QT when they see one. *Heart Rhythm* 2005; **2**(6): 569-74.

2. Chue AL, Moore RL, Cavey A, et al. Comparability of tympanic and oral mercury thermometers at high ambient temperatures. *BMC Res Notes* 2012; **5**: 356.

3. Lee SJ, Stepniewska K, Anstey N, et al. The relationship between the haemoglobin concentration and the haematocrit in Plasmodium falciparum malaria. *Malar J* 2008; **7**: 149.

4. World Health Organization. WHO Evidence Review Group on the Cardiotoxicity of Antimalarial Medicines. Geneva, Switzerland, 2017.

5. Stan Development Team. shinystan: Interactive Visual and Numerical Diagnostics and Posterior Analysis for Bayesian Models. 2017.

6. Abernethy DR, Wesche DL, Barbey JT, et al. Stereoselective halofantrine disposition and effect: concentration-related QTc prolongation. *Br J Clin Pharmacol* 2001; **51**(3): 231-7.

7. Ahmed R, Poespoprodjo JR, Syafruddin D, et al. Efficacy and safety of intermittent preventive treatment and intermittent screening and treatment versus single screening and treatment with dihydroartemisinin-piperaquine for the control of malaria in pregnancy in Indonesia: a cluster-randomised, open-label, superiority trial. *Lancet Infect Dis* 2019; **19**(9): 973-87.

8. Baiden R, Oduro A, Halidou T, et al. Prospective observational study to evaluate the clinical safety of the fixed-dose artemisinin-based combination Eurartesim(R) (dihydroartemisinin/piperaquine), in public health facilities in Burkina Faso, Mozambique, Ghana, and Tanzania. *Malar J* 2015; **14**: 160.

9. Bassat Q, Mulenga M, Tinto H, et al. Dihydroartemisinin-piperaquine and artemether-lumefantrine for treating uncomplicated malaria in African children: a randomised, non-inferiority trial. *PLoS One* 2009; **4**(11): e7871.

10. Bassi PU, Onyeji CO, Ukponmwan OE. Effects of tetracycline on the pharmacokinetics of halofantrine in healthy volunteers. *Br J Clin Pharmacol* 2004; **58**(1): 52-5.

11. Darpo B, Ferber G, Siegl P, et al. Evaluation of the QT effect of a combination of piperaquine and a novel anti-malarial drug candidate OZ439, for the treatment of uncomplicated malaria. *Br J Clin Pharmacol* 2015; **80**(4): 706-15.

12. Funck-Brentano C, Bacchieri A, Valentini G, et al. Effects of Dihydroartemisinin-Piperaquine Phosphate and Artemether-Lumefantrine on QTc Interval Prolongation. *Sci Rep* 2019; **9**(1): 777.

13. Hanboonkunupakarn B, Ashley EA, Jittamala P, et al. Open-label crossover study of primaquine and dihydroartemisinin-piperaquine pharmacokinetics in healthy adult thai subjects. *Antimicrob Agents Chemother* 2014; **58**(12): 7340-6.

14. Hanboonkunupakarn B, van der Pluijm RW, Hoglund R, et al. Sequential Open-Label Study of the Safety, Tolerability, and Pharmacokinetic Interactions between Dihydroartemisinin-Piperaquine and Mefloquine in Healthy Thai Adults. *Antimicrob Agents Chemother* 2019; **63**(8).

15. Kredo T, Mauff K, Van der Walt JS, et al. Interaction between artemether-lumefantrine and nevirapine-based antiretroviral therapy in HIV-1-infected patients. *Antimicrob Agents Chemother* 2011; **55**(12): 5616-23.

16. Kredo T, Mauff K, Workman L, et al. The interaction between artemether-lumefantrine and lopinavir/ritonavir-based antiretroviral therapy in HIV-1 infected patients. *BMC Infect Dis* 2016; **16**: 30.

17. Krudsood S, Looareesuwan S, Tangpukdee N, et al. New fixed-dose artesunate-mefloquine formulation against multidrug-resistant Plasmodium falciparum in adults: a comparative phase IIb safety and pharmacokinetic study with standard-dose nonfixed artesunate plus mefloquine. *Antimicrob Agents Chemother* 2010; **54**(9): 3730-7.

18. Macintyre F, Adoke Y, Tiono AB, et al. A randomised, double-blind clinical phase II trial of the efficacy, safety, tolerability and pharmacokinetics of a single dose combination treatment with artefenomel and piperaquine in adults and children with uncomplicated Plasmodium falciparum malaria. *BMC Med* 2017; **15**(1): 181.

19. Mytton OT, Ashley EA, Peto L, et al. Short report: Electrocardiographic safety evaluation of dihydroartemisinin-piperaquine in the treatment of uncomplicated falciparum malaria. *Am J Trop Med Hyg* 2007; **77**(3): 447-50.

20. Navaratnam V, Ramanathan S, Wahab MS, et al. Tolerability and pharmacokinetics of non-fixed and fixed combinations of artesunate and amodiaquine in Malaysian healthy normal volunteers. *Eur J Clin Pharmacol* 2009; **65**(8): 809-21.

21. Ndiaye JL, Faye B, Gueye A, et al. Repeated treatment of recurrent uncomplicated Plasmodium falciparum malaria in Senegal with fixed-dose artesunate plus amodiaquine versus fixed-dose artemether plus lumefantrine: a randomized, open-label trial. *Malar J* 2011; **10**: 237.

22. Nosten F, ter Kuile FO, Luxemburger C, et al. Cardiac effects of antimalarial treatment with halofantrine. *Lancet* 1993; **341**(8852): 1054-6.

23. Ogutu B, Juma E, Obonyo C, et al. Fixed dose artesunate amodiaquine - a phase IIb, randomized comparative trial with non-fixed artesunate amodiaquine. *Malar J* 2014; **13**: 498.

24. Price RN, Nosten F, Luxemburger C, et al. Artesunate versus artemether in combination with mefloquine for the treatment of multidrug-resistant falciparum malaria. *Trans R Soc Trop Med Hyg* 1995; **89**(5): 523-7.

25. Price RN, Nosten F, Luxemburger C, et al. Artesunate/mefloquine treatment of multi-drug resistant falciparum malaria. *Trans R Soc Trop Med Hyg* 1997; **91**(5): 574-7.

26. Price R, Luxemburger C, van Vugt M, et al. Artesunate and mefloquine in the treatment of uncomplicated multidrug-resistant hyperparasitaemic falciparum malaria. *Trans R Soc Trop Med Hyg* 1998; **92**(2): 207-11.

27. Price R, van Vugt M, Nosten F, et al. Artesunate versus artemether for the treatment of recrudescent multidrug-resistant falciparum malaria. *Am J Trop Med Hyg* 1998; **59**(6): 883-8.

28. Kakuru A, Jagannathan P, Muhindo MK, et al. Dihydroartemisinin–Piperaquine for the Prevention of Malaria in Pregnancy. *New England Journal of Medicine* 2016; **374**(10): 928-39.

29. Natureeba P, Kakuru A, Muhindo M, et al. Intermittent Preventive Treatment with Dihydroartemisinin-piperaquine for the Prevention of Malaria among HIV-infected Pregnant Women. *J Infect Dis* 2017.

30. Pukrittayakamee S, Tarning J, Jittamala P, et al. Pharmacokinetic interactions between primaquine and chloroquine. *Antimicrobial agents and chemotherapy* 2014; **58**(6): 3354-9.

31. Siqueira AM, Alencar AC, Melo GC, et al. Fixed-Dose Artesunate–Amodiaquine Combination vs Chloroquine for Treatment of Uncomplicated Blood Stage P. vivax Infection in the Brazilian Amazon: An Open-Label Randomized, Controlled Trial. *Clinical Infectious Diseases: An Official Publication of the Infectious Diseases Society of America* 2017; **64**(2): 166-74.

32. Toure OA, Rulisa S, Anvikar AR, et al. Efficacy and safety of fixed dose combination of arterolane maleate and piperaquine phosphate dispersible tablets in paediatric patients with acute uncomplicated Plasmodium falciparum malaria: a phase II, multicentric, open-label study. *Malaria journal* 2015; **14**: 469.

33. Toure OA, Valecha N, Tshefu AK, et al. A Phase 3, Double-Blind, Randomized Study of Arterolane Maleate-Piperaquine Phosphate vs Artemether-Lumefantrine for Falciparum Malaria in Adolescent and Adult Patients in Asia and Africa. *Clinical infectious diseases : an official publication of the Infectious Diseases Society of America* 2016; **62**(8): 964-71.

34. Tran TH, Day NP, Nguyen HP, et al. A controlled trial of artemether or quinine in Vietnamese adults with severe falciparum malaria. *N Engl J Med* 1996; **335**(2): 76-83.

35. Valecha N, Phyo AP, Mayxay M, et al. An open-label, randomised study of dihydroartemisinin-piperaquine versus artesunate-mefloquine for falciparum malaria in Asia. *PLoS One* 2010; **5**(7): e11880.

36. Valecha N, Krudsood S, Tangpukdee N, et al. Arterolane maleate plus piperaquine phosphate for treatment of uncomplicated Plasmodium falciparum malaria: a comparative, multicenter, randomized clinical trial. *Clin Infect Dis* 2012; **55**(5): 663-71.

37. Valecha N, Savargaonkar D, Srivastava B, et al. Comparison of the safety and efficacy of fixed-dose combination of arterolane maleate and piperaquine phosphate with chloroquine in acute, uncomplicated Plasmodium vivax malaria: a phase III, multicentric, open-label study. *Malaria journal* 2016; **15**: 42.

38. van Vugt M, Wilairatana P, Gemperli B, et al. Efficacy of six doses of artemether-lumefantrine (benflumetol) in multidrug-resistant Plasmodium falciparum malaria. *The American journal of tropical medicine and hygiene* 1999; **60**(6): 936-42.

39. van Vugt M, Looareesuwan S, Wilairatana P, et al. Artemether-lumefantrine for the treatment of multidrug-resistant falciparum malaria. *Trans R Soc Trop Med Hyg* 2000; **94**(5): 545-8.

40. Sagara I, Beavogui AH, Zongo I, et al. Safety and efficacy of re-treatments with pyronaridine-artesunate in African patients with malaria: a substudy of the WANECAM randomised trial. *The Lancet Infectious Diseases* 2016; **16**(2): 189-98.

41. West African Network for Clinical Trials of Antimalarial D. Pyronaridine-artesunate or dihydroartemisinin-piperaquine versus current first-line therapies for repeated treatment of uncomplicated malaria: a randomised, multicentre, open-label, longitudinal, controlled, phase 3b/4 trial. *Lancet* 2018; **391**(10128): 1378-90.

42. White NJ, Miller KD, Churchill FC, et al. Chloroquine treatment of severe malaria in children. Pharmacokinetics, toxicity, and new dosage recommendations. *N Engl J Med* 1988; **319**(23): 1493-500.

43. Abdulla S, Sagara I, Borrmann S, et al. Efficacy and safety of artemether-lumefantrine dispersible tablets compared with crushed commercial tablets in African infants and children with uncomplicated malaria: a randomised, single-blind, multicentre trial. *Lancet* 2008; **372**(9652): 1819-27.

44. Abdulla S, Amuri B, Kabanywanyi AM, et al. Early clinical development of artemether-lumefantrine dispersible tablet: palatability of three flavours and bioavailability in healthy subjects. *Malar J* 2010; **9**: 253.

45. Alecrim MG, Lacerda MV, Mourao MP, et al. Successful treatment of Plasmodium falciparum malaria with a six-dose regimen of artemether-lumefantrine versus quinine-doxycycline in the Western Amazon region of Brazil. *The American journal of tropical medicine and hygiene* 2006; **74**(1): 20-5.

46. Assimadi JK, Gbadoe AD, Agbodjan-Djossou O, et al. [Treatment of cerebral malaria in African children by intravenous quinine: comparison of a loading dose regimen to a regimen without a loading dose]. *Archives de pediatrie : organe officiel de la Societe francaise de pediatrie* 2002; **9**(6): 587-94.

47. Benjamin JM, Moore BR, Salman S, et al. Population Pharmacokinetics, Tolerability, and Safety of Dihydroartemisinin-Piperaquine and Sulfadoxine-Pyrimethamine-Piperaquine in Pregnant and Nonpregnant Papua New Guinean Women. *Antimicrobial Agents and Chemotherapy* 2015; **59**(7): 4260-71.

48. Bigira V, Kapisi J, Clark TD, et al. Protective efficacy and safety of three antimalarial regimens for the prevention of malaria in young Ugandan children: a randomized controlled trial. *PLoS Med* 2014; **11**(8): e1001689.

49. Bindschedler M, Lefevre G, Ezzet F, Schaeffer N, Meyer I, Thomsen MS. Cardiac effects of co-artemether (artemether/lumefantrine) and mefloquine given alone or in combination to healthy volunteers. *European journal of clinical pharmacology* 2000; **56**(5): 375-81.

50. Bindschedler M, Lefevre G, Degen P, Sioufi A. Comparison of the cardiac effects of the antimalarials co-artemether and halofantrine in healthy participants. *Am J Trop Med Hyg* 2002; **66**(3): 293-8.

51. Bouchaud O, Monlun E, Muanza K, et al. Atovaquone plus proguanil versus halofantrine for the treatment of imported acute uncomplicated Plasmodium falciparum malaria in non-immune adults: a randomized comparative trial. *Am J Trop Med Hyg* 2000; **63**(5-6): 274-9.

52. Bouyou-Akotet MK, Ramharter M, Ngoungou EB, et al. Efficacy and safety of a new pediatric artesunate-mefloquine drug formulation for the treatment of uncomplicated falciparum malaria in Gabon. *Wiener klinische Wochenschrift* 2010; **122**(5-6): 173-8.

53. Bunnag D, Harinasuta T, Looareesuwan S, et al. A combination of quinine, quinidine and cinchonine (LA 40221) in the treatment of chloroquine resistant falciparum malaria in Thailand: two double-blind trials. *Transactions of the Royal Society of Tropical Medicine and Hygiene* 1989; **83**(1): 66.

54. Cao XT, Bethell DB, Pham TP, et al. Comparison of artemisinin suppositories, intramuscular artesunate and intravenous quinine for the treatment of severe childhood malaria. *Transactions of the Royal Society of Tropical Medicine and Hygiene* 1997; **91**(3): 335-42.

55. Haroon N, Amichandwala K, Solu MG. Comparative efficacy of quinine and artesunate in the treatment of severe malaria: A randomized controlled trial. *JK Science* 2005; **7**(1): 32-5.

56. Hien TT, Hanpithakpong W, Truong NT, et al. Orally formulated artemisinin in healthy fasting Vietnamese male subjects: a randomized, four-sequence, open-label, pharmacokinetic crossover study. *Clinical therapeutics* 2011; **33**(5): 644-54.

57. Jittamala P, Pukrittayakamee S, Ashley EA, et al. Pharmacokinetic interactions between primaquine and pyronaridine-artesunate in healthy adult Thai subjects. 2015; **59**(1): 505-13.

58. Kakuda TN, DeMasi R, van Delft Y, Mohammed P. Pharmacokinetic interaction between etravirine or darunavir/ritonavir and artemether/lumefantrine in healthy volunteers: a two-panel, two-way, two-period, randomized trial. *Antimicrobial agents and chemotherapy* 2013; **14**(7): 421-9.

59. Karbwang J, Bangchang KN, Bunnag D, Harinasuta T. Pharmacokinetics and pharmacodynamics of mefloquine in Thai patients with acute falciparum malaria. *Bull World Health Organ* 1991; **69**(2): 207-12.

60. Karbwang J, Na Bangchang K, Back DJ, Bunnag D, Rooney W. Effect of tetracycline on mefloquine pharmacokinetics in Thai males. *European journal of clinical pharmacology* 1992; **43**(5): 567-9.

61. Karbwang J, Sukontason K, Rimchala W, et al. Preliminary report: a comparative clinical trial of artemether and quinine in severe falciparum malaria. *The Southeast Asian journal of tropical medicine and public health* 1992; **23**(4): 768-72.

62. Karbwang J, Davis TM, Looareesuwan S, Molunto P, Bunnag D, White NJ. A comparison of the pharmacokinetic and pharmacodynamic properties of quinine and quinidine in healthy Thai males. *British journal of clinical pharmacology* 1993; **35**(3): 265-71.

63. Karbwang J, Tin T, Rimchala W, et al. Comparison of artemether and quinine in the treatment of severe falciparum malaria in south-east Thailand. *Transactions of the Royal Society of Tropical Medicine and Hygiene* 1995; **89**(6): 668-71.

64. Karbwang J, Na-Bangchang K, Thanavibul A, Laothavorn P, Ditta-in M, Harinasuta T. A comparative clinical trial of artemether and the sequential regimen of artemether-mefloquine in multidrug resistant falciparum malaria. *The Journal of antimicrobial chemotherapy* 1995; **36**(6): 1079-83.

65. Karbwang J, Na-Bangchang K, Thanavibul A, Ditta-in M, Harinasuta T. A comparative clinical trial of two different regimens of artemether plus mefloquine in multidrug resistant falciparum malaria. *Transactions of the Royal Society of Tropical Medicine and Hygiene* 1995; **89**(3): 296-8.

66. Karbwang J, Laothavorn P, Sukontason K, et al. Effect of artemether on electrocardiogram in severe falciparum malaria. *The Southeast Asian journal of tropical medicine and public health* 1997; **28**(3): 472-5.

67. Kayentao K, Doumbo OK, Penali LK, et al. Pyronaridine-artesunate granules versus artemether-lumefantrine crushed tablets in children with Plasmodium falciparum malaria: a randomized controlled trial. *Malaria journal* 2012; **11**: 364.

68. Khan SJ, Amanullah, Shah N, Ali M. Efficacy of loading versus standard doses of quinine in cerebral malaria. *Rawal Med J* 2011; **36**(2): 86-8.

69. Kinde-Gazard D, Ogouyemi-Hounto A, Capo-Chichi L, Gbaguidi J, Massougbodji A. [A randomized clinical trial comparing the effectiveness and tolerability of artemisinine-naphthoquine (Arco(R)) and artemether-lumefantrine (Coartem(R)) in the treatment of uncomplicated malaria in Benin]. *Bulletin de la Societe de pathologie exotique (1990)* 2012; **105**(3): 208-14.

70. Kshirsagar NA, Gogtay NJ, Moorthy NS, et al. A randomized, double-blind, parallel-group, comparative safety, and efficacy trial of oral co-artemether versus oral chloroquine in the treatment of acute uncomplicated Plasmodium falciparum malaria in adults in India. *Am J Trop Med Hyg* 2000; **62**(3): 402-8.

71. Laman M, Moore BR, Benjamin JM, et al. Artemisinin-naphthoquine versus artemether-lumefantrine for uncomplicated malaria in Papua New Guinean children: an open-label randomized trial. *PLoS medicine* 2014; **11**(12): e1001773.

72. Lefevre G, Looareesuwan S, Treeprasertsuk S, et al. A clinical and pharmacokinetic trial of six doses of artemether-lumefantrine for multidrug-resistant Plasmodium falciparum malaria in Thailand. *The American journal of tropical medicine and hygiene* 2001; **64**(5-6): 247-56.

73. Lefevre G, Carpenter P, Souppart C, Schmidli H, McClean M, Stypinski D. Pharmacokinetics and electrocardiographic pharmacodynamics of artemether-lumefantrine (Riamet) with concomitant administration of ketoconazole in healthy subjects. *British journal of clinical pharmacology* 2002; **54**(5): 485-92.

74. Lefevre G, Carpenter P, Souppart C, et al. Interaction trial between artemether-lumefantrine (Riamet) and quinine in healthy subjects. *Journal of clinical pharmacology* 2002; **42**(10): 1147-58.

75. Lefevre G, Bhad P, Jain JP, et al. Evaluation of two novel tablet formulations of artemether-lumefantrine (Coartem) for bioequivalence in a randomized, open-label, two-period study. *Malaria journal* 2013; **12**: 312.

76. Liu Y, Hu C, Liu G, et al. A replicate designed bioequivalence study to compare two fixed-dose combination products of artesunate and amodiaquine in healthy chinese volunteers. *Antimicrob Agents Chemother* 2014; **58**(10): 6009-15.

77. Llanos-Cuentas A, Lacerda MV, Rueangweerayut R, et al. Tafenoquine plus chloroquine for the treatment and relapse prevention of Plasmodium vivax malaria (DETECTIVE): a multicentre, double-blind, randomised, phase 2b dose-selection study. *Lancet (London, England)* 2014; **383**(9922): 1049-58.

78. Lon C, Manning JE, Vanachayangkul P, et al. Efficacy of two versus three-day regimens of dihydroartemisinin-piperaquine for uncomplicated malaria in military personnel in northern Cambodia: an open-label randomized trial. *PloS one* 2014; **9**(3): e93138.

79. Manning J, Vanachayangkul P, Lon C, et al. Randomized, double-blind, placebo-controlled clinical trial of a two-day regimen of dihydroartemisinin-piperaquine for malaria prevention halted for concern over prolonged corrected QT interval. *Antimicrob Agents Chemother* 2014; **58**(10): 6056-67.

80. Massougbodji A, Kone M, Kinde-Gazard D, Same-Ekobo A, Cambon N, Mueller EA. A randomized, double-blind study on the efficacy and safety of a practical three-day regimen with artesunate and mefloquine for the treatment of uncomplicated Plasmodium falciparum malaria in Africa. *Transactions of the Royal Society of Tropical Medicine and Hygiene* 2002; **96**(6): 655-9.

81. McGready R, Tan SO, Ashley EA, et al. A randomised controlled trial of artemether-lumefantrine versus artesunate for uncomplicated plasmodium falciparum treatment in pregnancy. *PLoS Med* 2008; **5**(12): e253.

82. Miller AK, Harrell E, Ye L, et al. Pharmacokinetic interactions and safety evaluations of coadministered tafenoquine and chloroquine in healthy subjects. *British journal of clinical pharmacology* 2013; **76**(6): 858-67.

83. Moore BR, Benjamin JM, Salman S, et al. Effect of coadministered fat on the tolerability, safety, and pharmacokinetic properties of dihydroartemisinin-piperaquine in Papua New Guinean children with uncomplicated malaria. *Antimicrobial agents and chemotherapy* 2014; **58**(10): 5784-94.

84. Morris CA, Lopez-Lazaro L, Jung D, et al. Drug-Drug Interaction Analysis of Pyronaridine/Artesunate and Ritonavir in Healthy Volunteers. *The American journal of tropical medicine and hygiene* 2012; **86**(3): 489-95.

85. Murphy S, English M, Waruiru C, et al. An open randomized trial of artemether versus quinine in the treatment of cerebral malaria in African children. *Transactions of the Royal Society of Tropical Medicine and Hygiene* 1996; **90**(3): 298-301.

86. Mutabingwa TK, Muze K, Ord R, et al. Randomized trial of artesunate+amodiaquine, sulfadoxine-pyrimethamine+amodiaquine, chlorproguanal-dapsone and SP for malaria in pregnancy in Tanzania. *PLoS One* 2009; **4**(4): e5138.

87. Mzayek F, Deng H, Mather FJ, et al. Randomized dose-ranging controlled trial of AQ-13, a candidate antimalarial, and chloroquine in healthy volunteers. *PLoS Clin Trials* 2007; **2**(1): e6.

88. Na-Bangchang K, Karbwang J, Palacios PA, Ubalee R, Saengtertsilapachai S, Wernsdorfer WH. Pharmacokinetics and bioequivalence evaluation of three commercial tablet formulations of mefloquine when given in combination with dihydroartemisinin in patients with acute uncomplicated falciparum malaria. *European journal of clinical pharmacology* 2000; **55**(10): 743-8.

89. Na-Bangchang K, Thanavibul A, Tippawangkosol P, Karbwang J. Pharmacokinetics of the four combination regimens of dihydroartemisinin/mefloquine in acute uncomplicated falciparum malaria. *The Southeast Asian journal of tropical medicine and public health* 2005; **36**(1): 23-33.

90. Nasveld PE, Edstein MD, Reid M, et al. Randomized, double-blind study of the safety, tolerability, and efficacy of tafenoquine versus mefloquine for malaria prophylaxis in nonimmune subjects. *Antimicrobial agents and chemotherapy* 2010; **54**(2): 792-8.

91. Nelwan EJ, Ekawati LL, Tjahjono B, et al. Randomized trial of primaquine hypnozoitocidal efficacy when administered with artemisinin-combined blood schizontocides for radical cure of Plasmodium vivax in Indonesia. *BMC Medicine* 2015; **13**: 294.

92. Newton PN, Chierakul W, Ruangveerayuth R, et al. A comparison of artesunate alone with combined artesunate and quinine in the parenteral treatment of acute falciparum malaria. *Transactions of the Royal Society of Tropical Medicine and Hygiene* 2001; **95**(5): 519-23.

93. Ngouesse B, Basco LK, Ringwald P, Keundjian A, Blackett KN. Cardiac effects of amodiaquine and sulfadoxine-pyrimethamine in malaria-infected African patients. *Am J Trop Med Hyg* 2001; **65**(6): 711-6.

94. Nosten F, Karbwang J, White NJ, et al. Mefloquine antimalarial prophylaxis in pregnancy: dose finding and pharmacokinetic study. *British journal of clinical pharmacology* 1990; **30**(1): 79-85.

95. Nosten F, ter Kuile F, Maelankiri L, et al. Mefloquine prophylaxis prevents malaria during pregnancy: a double-blind, placebo-controlled study. *J Infect Dis* 1994; **169**(3): 595-603.

96. Olliaro PL, Ramanathan S, Vaillant M, et al. Pharmacokinetics and Comparative Bioavailability of Artesunate and Mefloquine Administered Separately or as a Fixed Combination Product to Healthy Volunteers and Patients with Uncomplicated Plasmodium falciparum Malaria. *Journal of Bioequivalence & Bioavailability* 2010; **2**(3): 59-66.

97. Omoruyi SI, Onyeji CO, Daniyan MO. Effects of prior administration of amodiaquine on the disposition of halofantrine in healthy volunteers. *Ther Drug Monit* 2007; **29**(2): 203-6.

98. Orrell C, Little F, Smith P, et al. Pharmacokinetics and tolerability of artesunate and amodiaquine alone and in combination in healthy volunteers. *Eur J Clin Pharmacol* 2008; **64**(7): 683-90.

99. Piola P, Nabasumba C, Turyakira E, et al. Efficacy and safety of artemether-lumefantrine compared with quinine in pregnant women with uncomplicated Plasmodium falciparum malaria: an open-label, randomised, non-inferiority trial. *The Lancet Infectious diseases* 2010; **10**(11): 762-9.

100. Poravuth Y, Socheat D, Rueangweerayut R, et al. Pyronaridine-artesunate versus chloroquine in patients with acute Plasmodium vivax malaria: a randomized, double-blind, non-inferiority trial. *PLoS One* 2011; **6**(1): e14501.

101. Pyar KP, Myint WW, Kyaw MP, et al. Comparison of efficacy and safety of different brands of oral artesunate plus mefloquine in uncomplicated falciparum malaria in adults. *Myanmar Heal Sci Res J* 2007; **21**: 78-82.

102. Pyar KP, Myint WW, Kyaw MP, Zin T, Than M. Efficacy and safety of artemisinin-piperaquine (Artequick) compared to dihydroartemisinin-piperaquine (Artekin) in uncomplicated falciparum malaria in adults. *Myanmar Health Sciences Research Journal* 2009; **21**(2): 78-82.

103. Restrepo M, Botero D, Marquez RE, Boudreau EF, Navaratnam V. A clinical trial with halofantrine on patients with falciparum malaria in Colombia. *Bulletin of the World Health Organization* 1996; **74**(6): 591-7.

104. Rueangweerayut R, Phyo AP, Uthaisin C, et al. Pyronaridine-artesunate versus mefloquine plus artesunate for malaria. *N Engl J Med* 2012; **366**(14): 1298-309.

105. Sabchareon A, Chongsuphajaisiddhi T, Sinhasivanon V, Chanthavanich P, Attanath P. In vivo and in vitro responses to quinine and quinidine of Plasmodium falciparum. *Bulletin of the World Health Organization* 1988; **66**(3): 347-52.

106. Song J, Socheat D, Tan B, et al. Randomized trials of artemisinin-piperaquine, dihydroartemisinin-piperaquine phosphate and artemether-lumefantrine for the treatment of multi-drug resistant falciparum malaria in Cambodia-Thailand border area. *Malaria journal* 2011; **10**: 231.

107. Sowunmi A, Salako LA, Laoye OJ, Aderounmu AF. Combination of quinine, quinidine and cinchonine for the treatment of acute falciparum malaria: correlation with the susceptibility of Plasmodium falciparum to the cinchona alkaloids in vitro. *Transactions of the Royal Society of Tropical Medicine and Hygiene* 1990; **84**(5): 626-9.

108. Staedke SG, Maiteki-Sebuguzi C, Rehman AM, et al. Assessment of community-level effects of intermittent preventive treatment for malaria in schoolchildren in Jinja, Uganda (START-IPT trial): a cluster-randomised trial. *Lancet Glob Health* 2018; **6**(6): e668-e79.

109. Supan C, Mombo-Ngoma G, Kombila M, et al. Phase 2a, Open-Label, 4-Escalating-Dose, Randomized Multicenter Study Evaluating the Safety and Activity of Ferroquine (SSR97193) Plus Artesunate, versus Amodiaquine Plus Artesunate, in African Adult Men with Uncomplicated Plasmodium falciparum Malaria. *Am J Trop Med Hyg* 2017; **97**(2): 514-25.

110. Taylor TE, Wills BA, Courval JM, Molyneux ME. Intramuscular artemether vs intravenous quinine: an open, randomized trial in Malawian children with cerebral malaria. *Tropical medicine & international health : TM & IH* 1998; **3**(1): 3-8.

111. Thapa S, Hollander J, Linehan M, et al. Comparison of artemether-lumefantrine with sulfadoxine-pyrimethamine for the treatment of uncomplicated falciparum malaria in eastern Nepal. *The American journal of tropical medicine and hygiene* 2007; **77**(3): 423-30.

112. Thuma PE, Bhat GJ, Mabeza GF, et al. A randomized controlled trial of artemotil (beta-arteether) in Zambian children with cerebral malaria. *The American journal of tropical medicine and hygiene* 2000; **62**(4): 524-9.

113. Tjitra E, Hasugian AR, Siswantoro H, et al. Efficacy and safety of artemisinin-naphthoquine versus dihydroartemisinin-piperaquine in adult patients with uncomplicated malaria: a multi-centre study in Indonesia. *Malaria journal* 2012; **11**: 153.

114. Touze JE, Heno P, Fourcade L, et al. The effects of antimalarial drugs on ventricular repolarization. *Am J Trop Med Hyg* 2002; **67**(1): 54-60.

115. Trung TN, Tan B, Van Phuc D, Song JP. A randomized, controlled trial of artemisinin-piperaquine vs dihydroartemisinin-piperaquine phosphate in treatment of falciparum malaria. *Chinese journal of integrative medicine* 2009; **15**(3): 189-92.

116. Tshefu AK, Gaye O, Kayentao K, et al. Efficacy and safety of a fixed-dose oral combination of pyronaridine-artesunate compared with artemether-lumefantrine in children and adults with uncomplicated Plasmodium falciparum malaria: a randomised non-inferiority trial. *Lancet* 2010; **375**(9724): 1457-67.

117. van Agtmael M, Bouchaud O, Malvy D, et al. The comparative efficacy and tolerability of CGP 56697 (artemether + lumefantrine) versus halofantrine in the treatment of uncomplicated falciparum malaria in travellers returning from the Tropics to The Netherlands and France. *Int J Antimicrob Agents* 1999; **12**(2): 159-69.

118. van Hensbroek MB, Kwiatkowski D, van den Berg B, Hoek FJ, van Boxtel CJ, Kager PA. Quinine pharmacokinetics in young children with severe malaria. *The American journal of tropical medicine and hygiene* 1996; **54**(3): 237-42.

119. Walker O, Salako LA, Omokhodion SI, Sowunmi A. An open randomized comparative study of intramuscular artemether and intravenous quinine in cerebral malaria in children. *Transactions of the Royal Society of Tropical Medicine and Hygiene* 1993; **87**(5): 564-6.

120. Adjei GO, Oduro-Boatey C, Rodrigues OP, et al. Electrocardiographic study in Ghanaian children with uncomplicated malaria, treated with artesunate-amodiaquine or artemether-lumefantrine. *Malar J* 2012; **11**: 420.

121. Auprayoon P, Sukontason K, Na-Bangchang K, Banmairuroi V, Molunto P, Karbwang J. Pharmacokinetics of quinine in chronic liver disease. *British journal of clinical pharmacology* 1995; **40**(5): 494-7.

122. Bhatt KM, Samia BM, Bhatt SM, Wasunna KM. Efficacy and safety of an artesunate/mefloquine combination, (artequin) in the treatment of uncomplicated P. falciparum malaria in Kenya. *East African medical journal* 2006; **83**(5): 236-42.

123. Byakika-Kibwika P, Lamorde M, Lwabi P, et al. Cardiac Conduction Safety during Coadministration of Artemether-Lumefantrine and Lopinavir/Ritonavir in HIV-Infected Ugandan Adults. *Chemotherapy research and practice* 2011; **2011**: 393976.

124. Claessen FA, van Boxtel CJ, Perenboom RM, Tange RA, Wetsteijn JC, Kager PA. Quinine pharmacokinetics: ototoxic and cardiotoxic effects in healthy Caucasian subjects and in patients with falciparum malaria. *Tropical medicine & international health : TM & IH* 1998; **3**(6): 482-9.

125. Davis TM, White NJ, Looareesuwan S, Silamut K, Warrell DA. Quinine pharmacokinetics in cerebral malaria: predicted plasma concentrations after rapid intravenous loading using a two-compartment model. *Transactions of the Royal Society of Tropical Medicine and Hygiene* 1988; **82**(4): 542-7.

126. Davis TM, Supanaranond W, Pukrittayakamee S, et al. A safe and effective consecutive-infusion regimen for rapid quinine loading in severe falciparum malaria. *The Journal of infectious diseases* 1990; **161**(6): 1305-8.

127. Edwards G, Looareesuwan S, Davies AJ, Wattanagoon Y, Phillips RE, Warrell DA. Pharmacokinetics of chloroquine in Thais: plasma and red-cell concentrations following an intravenous infusion to healthy subjects and patients with Plasmodium vivax malaria. *Br J Clin Pharmacol* 1988; **25**(4): 477-85.

128. Falade C, Makanga M, Premji Z, Ortmann CE, Stockmeyer M, de Palacios PI. Efficacy and safety of artemether-lumefantrine (Coartem) tablets (six-dose regimen) in African infants and children with acute, uncomplicated falciparum malaria. *Transactions of the Royal Society of Tropical Medicine and Hygiene* 2005; **99**(6): 459-67.

129. Haider I, Humayun M, Badshah A. The effect of quinine on QT interval in patients in a tertiary care hospital. *Journal of Postgraduate Medical Institute* 2013; **27**(1): 20-5.

130. Hatz C, Soto J, Nothdurft HD, et al. Treatment of acute uncomplicated falciparum malaria with artemether-lumefantrine in non-immune populations: A safety, efficacy, and pharmacokinetic study. *American Journal of Tropical Medicine and Hygiene* 2008; **78**(2): 241-7.

131. Hombhanje FW, Kereu RK, Bulungol P, Paika R. Halofantrine in the treatment of uncomplicated falciparum malaria with a three-dose regimen in Papua New Guinea: a preliminary report. *Papua and New Guinea medical journal* 1998; **41**(1): 23-9.

132. Jaspers CA, Hopperus Buma AP, van Thiel PP, van Hulst RA, Kager PA. Tolerance of mefloquine chemoprophylaxis in Dutch military personnel. *The American journal of tropical medicine and hygiene* 1996; **55**(2): 230-4.

133. Karbwang J, Thanavibul A, Molunto P, Na Bangchang K. The pharmacokinetics of quinine in patients with hepatitis. *British journal of clinical pharmacology* 1993; **35**(4): 444-6.

134. Karunajeewa H, Lim C, Hung TY, et al. Safety evaluation of fixed combination piperaquine plus dihydroartemisinin (Artekin) in Cambodian children and adults with malaria. *British journal of clinical pharmacology* 2004; **57**(1): 93-9.

135. Khan MZ, Isani Z, Ahmed TM, et al. Efficacy and safety of halofantrine in Pakistani children and adults with malaria caused by P. falciparum and P. vivax. *The Southeast Asian journal of tropical medicine and public health* 2006; **37**(4): 613-8.

136. Krishna S, ter Kuile F, Supanaranond W, et al. Pharmacokinetics, efficacy and toxicity of parenteral halofantrine in uncomplicated malaria. *Br J Clin Pharmacol* 1993; **36**(6): 585-91.

137. Lavallée I, Marc E, Moulin F, Treluyer JM, Imbert P, Gendrel D. Cardiac rhythm disturbances and prolongation of the QTc interval with halofantrine. *Arch Pediatr* 2001; **8**(8): 795-800.

138. Mansor SM, Taylor TE, McGrath CS, et al. The safety and kinetics of intramuscular quinine in Malawian children with moderately severe falciparum malaria. *Transactions of the Royal Society of Tropical Medicine and Hygiene* 1990; **84**(4): 482-7.

139. Matson PA, Luby SP, Redd SC, Rolka HR, Meriwether RA. Cardiac effects of standard-dose halofantrine therapy. *The American journal of tropical medicine and hygiene* 1996; **54**(3): 229-31.

140. Minodier P, Noel G, Salles M, et al. [Mefloquine versus halofantrine in children suffering from acute uncomplicated falciparum malaria]. *Archives de pediatrie : organe officiel de la Societe francaise de pediatrie* 2005; **12 Suppl 1**: S67-71.

141. Monlun E, Le Metayer P, Szwandt S, et al. Cardiac complications of halofantrine: a prospective study of 20 patients. *Trans R Soc Trop Med Hyg* 1995; **89**(4): 430-3.

142. Mra R, Myint P, Shwe T. Electrocardiographic effects of quinine and quinidine in the treatment of falciparum malaria. *Myanmar Heal Sci Res J* 1991; **3**: 1-5.

143. Na-Bangchang K, Limpaibul L, Thanavibul A, Tan-Ariya P, Karbwang J. The pharmacokinetics of chloroquine in healthy Thai subjects and patients with Plasmodium vivax malaria. *Br J Clin Pharmacol* 1994; **38**(3): 278-81.

144. Nyunt MM, Lu Y, El-Gasim M, Parsons TL, Petty BG, Hendrix CW. Effects of ritonavir-boosted lopinavir on the pharmacokinetics of quinine. *Clinical pharmacology and therapeutics* 2012; **91**(5): 889-95.

145. Ogunkunle OO, Fehintola FA, Ogungbamigbe T, Falade CO. Comparative cardiac effects of chlorproguanil/dapsone and chloroquine during treatment of acute uncomplicated falciparum malaria infection in Nigerian children. *Afr J Biomed Res* 2011; **14**(3): 161-7.

146. Roggelin L, Pelletier D, Hill JN, et al. Disease-associated QT-shortage versus quinine associated QT-prolongation: age dependent ECG-effects in Ghanaian children with severe malaria. *Malar J* 2014; **13**: 219.

147. Sowunmi A, Falade CO, Oduola AM, et al. Cardiac effects of halofantrine in children suffering from acute uncomplicated falciparum malaria. *Transactions of the Royal Society of Tropical Medicine and Hygiene* 1998; **92**(4): 446-8.

148. Stein DS, Jain JP, Kangas M, et al. Open-label, single-dose, parallel-group study in healthy volunteers to determine the drug-drug interaction potential between KAE609 (cipargamin) and piperaquine. *Antimicrobial agents and chemotherapy* 2015; **59**(6): 3493-500.

149. Sukontason K, Karbwang J, Rimchala W, et al. Plasma quinine concentrations in falciparum malaria with acute renal failure. *Tropical medicine & international health : TM & IH* 1996; **1**(2): 236-42.

150. Supanaranond W, Suputtamongkol Y, Davis TM, et al. Lack of a significant adverse cardiovascular effect of combined quinine and mefloquine therapy for uncomplicated malaria. *Transactions of the Royal Society of Tropical Medicine and Hygiene* 1997; **91**(6): 694-6.

151. Touze JE, Bernard J, Keundjian A, et al. Electrocardiographic changes and halofantrine plasma level during acute falciparum malaria. *Am J Trop Med Hyg* 1996; **54**(3): 225-8.

152. von Seidlein L, Jaffar S, Greenwood B. Prolongation of the QTc interval in African children treated for falciparum malaria. *Am J Trop Med Hyg* 1997; **56**(5): 494-7.

153. Win K, Than M, Thwe Y. Comparison of combinations of parenteral artemisinin derivatives plus oral mefloquine with intravenous quinine plus oral tetracycline for treating cerebral malaria. *Bulletin of the World Health Organization* 1992; **70**(6): 777-82.
